# Repurposing Drugs for Inhibition against ALDH2 via a 2D/3D Ligand-Based Similarity Search and Molecular Simulation

**DOI:** 10.3390/molecules28217325

**Published:** 2023-10-29

**Authors:** Wanyun Jiang, Junzhao Chen, Puyu Zhang, Nannan Zheng, Le Ma, Yongguang Zhang, Haiyang Zhang

**Affiliations:** Department of Biological Science and Engineering, School of Chemistry and Biological Engineering, University of Science and Technology Beijing, Beijing100083, China

**Keywords:** drug repurposing, ligand-based virtual screening, substance use disorder, receptor–ligand interactions

## Abstract

Aldehyde dehydrogenase-2 (ALDH2) is a crucial enzyme participating in intracellular aldehyde metabolism and is acknowledged as a potential therapeutic target for the treatment of alcohol use disorder and other addictive behaviors. Using previously reported ALDH2 inhibitors of Daidzin, CVT-10216, and CHEMBL114083 as reference molecules, here we perform a ligand-based virtual screening of world-approved drugs via 2D/3D similarity search methods, followed by the assessments of molecular docking, toxicity prediction, molecular simulation, and the molecular mechanics Poisson–Boltzmann surface area (MM–PBSA) analysis. The 2D molecular fingerprinting of ECFP4 and FCFP4 and 3D molecule-shape-based USRCAT methods show good performances in selecting compounds with a strong binding behavior with ALDH2. Three compounds of Zeaxanthin (*q* = 0), Troglitazone (*q* = 0), and Sequinavir (*q* = +1 *e*) are singled out as potential inhibitors; Zeaxanthin can only be hit via USRCAT. These drugs displayed a stronger binding strength compared to the reported potent inhibitor CVT-10216. Sarizotan (*q* = +1 *e*) and Netarsudil (*q* = 0/+1 *e*) displayed a strong binding strength with ALDH2 as well, whereas they displayed a shallow penetration into the substrate-binding tunnel of ALDH2 and could not fully occupy it. This likely left a space for substrate binding, and thus they were not ideal inhibitors. The MM–PBSA results indicate that the selected negatively charged compounds from the similarity search and Vina scoring are thermodynamically unfavorable, mainly due to electrostatic repulsion with the receptor (*q* = −6 *e* for ALDH2). The electrostatic attraction with positively charged compounds, however, yielded very strong binding results with ALDH2. These findings reveal a deficiency in the modeling of electrostatic interactions (in particular, between charged moieties) in the virtual screening via the 2D/3D similarity search and molecular docking with the Vina scoring system.

## 1. Introduction

Substance use disorder is a disease characterized by the uncontrolled intake of certain addictive substances that affects the human brain, psychology, and behavior [1]. These substances are able to elicit pleasurable sensations by strongly stimulating the brain’s reward system. Alcohol use disorder (AUD), for instance, is the most widespread addictive behavior. The toxic aldehydes (acetaldehyde) produced by alcohol (ethanol) metabolism can damage the morphology and function of proteins [2], DNA [3], organs [4], and tissues [5], leading to serious health issues. For example, it may result in alcoholic hepatitis, liver cirrhosis, liver cancer, or other liver diseases [6,7]; moreover, it increases the potential risk of neurodegenerative disorders, such as neuroinflammation, movement disorders, and cognitive dysfunction [8,9]. The types of withdrawal syndrome associated with alcohol include anxiety, limbs tremor, emesis, autonomic nervous system hyperfunction, and possibly more. This explains why it may be simple to develop an addiction but is more challenging to quit [10].

In the human body, acetaldehyde dehydrogenases (ALDHs) play a critical role in the metabolism of aldehydes, converting them into non-toxic carboxylic acids and related derivatives. Of the 19 members of the human ALDH superfamily, ALDH1A1, ALDH1B1, and ALDH2 are the three enzymes most closely involved in acetaldehyde metabolism [11]. Due to the extremely low *k*_m_ (*k*_m_ < 1 μM) levels and the wide distribution in various tissues, ALDH2 is the most effective one to oxidize and eliminate endogenous and exogenous aldehydes irreversibly [12]. Approximately 40% of Asians have an ALDH2 gene polymorphism, and the mutant ALDH2 allele (ALDH2*2) encodes an enzyme with a low activity. Even with the presence of only one ALDH2*2 subunit, heterotetramer ALDH2 exhibits a loss in function [13,14]. Individuals with this genetic polymorphism likely suffer from negative physiological reactions (disulfiram-like reactions), including flushing, dizziness, and palpitations, mainly due to the acetaldehyde accumulation in their bodies after alcohol intake. These protective symptoms alert the drinker to avoid alcohol consumption and hence reduce the incidence of alcohol addiction [15]. Stress and anxiety are the major symptoms of addictive behavior. Studies on animals have shown that inhibiting ALDH2 enzyme activity can ease anxiety [16], decrease binge eating [17], and reduce the urge to seek out alcohol [18], cocaine [19], and methamphetamine [20]. This might be ascribed to the fact that ALDH2 inhibition suppresses the synthesis of drug-associated dopamine [19]. ALDH2 is therefore a crucial target for treating AUD and preventing a relapse.

Anti-craving drugs that are being developed for targeting ALDH2, opioid receptors, the GABA (*γ*-aminobutyric acid) receptor, and the NMDA (*N*-methyl-*d*-aspartate) receptor are becoming a highly sought-after field for basic and applied research. Baclofen, gabapentin, topiramate, and ondansetron are still under clinical research due to their side effects and non-specific alcohol abuse [21,22]. For the treatment of AUD, there are four clinical drugs: disulfiram, acamprosate, naltrexone, and nalmefene; the former three were approved by the U.S. Food and Drug Administration (FDA) and the latter was approved the European Medicines Agency (EMA) [22,23]. Only disulfiram targets ALDH2. However, disulfiram inhibits ALDH1 more effectively than ALDH2 and it has a global inhibitory effect on ALDH2 [24,25]. In a variety of organs, ALDH2 protects tissues from damage by reducing oxidative stress and removing toxic aldehydes [26,27,28,29]. Daidzin is a naturally occurring product and is a specific inhibitor of ALDH2 (IC_50_ = 0.08 μM) [30]. Keung et al. synthesized a number of isoflavone analogs, of which CHEMBL114083 (compound **20** in their work) showed the strongest inhibition (IC_50_ = 0.04 μM) [30]. They also crystallized the complex of ALDH2 with Daidzin at 2.4 Å (PDB code: 2VLE), offering a structural basis for the specificity and affinity of Daidzin against ALDH2 [31]. Inspired by this crystal structure, a highly effective ALDH2 inhibitor, CVT-10216 (IC_50_ = 0.029 μM), was proposed with a therapeutic potential to suppress heavy drinking and alcohol seeking [18]; it had the ability to inhibit ALDH2 reversibly while resulting in a minimal interference with other enzymes and receptors within the body [18].

Computer virtual screening has become an important solution for accelerating the search of lead compounds for new drug development and drug repurposing from a large-scale compound database. Based on the concept that similar molecular structures and/or shapes likely have similar physiological activities and biological functions, similarity search methods with 2D/3D molecular fingerprints and/or 3D molecular shapes have proved to be effective in structure-based virtual screening [32,33]. Other techniques, such as molecular docking and molecular simulation, can be combined with the 2D/3D similarity search for a high-throughput virtual screening in a parallel or hierarchical manner [34,35]. Using fusidic acid as a reference compound, Pavadai et al. performed the 2D/3D similarity-based virtual screening of steroid-type natural products and hit a few growth inhibitors of *Plasmodium falciparum* with a low IC_50_ and comparable ADME (absorption, distribution, metabolism, and excretion) properties to the reference compound [36]. The 2D and 3D similarity-based approach was also used to find potential inhibitors for diverse targets, such as human hexokinase II (HKII) [37], p53-MDM2 [38], and HIV-1 [39].

For the receptor ALDH2, however, there are few reports on the discovery of its potent inhibitors. Wang and coworkers identified five small-molecule inhibitors from a screening of a commercial chemical database (50,000 compounds), and their IC_50_ values ranged from 0.5 to 23 μM [40], higher than those for the isoflavone analogs mentioned above. Here, we chose three isoflavone analogs of Daidzin, CVT-10216, and CHEMBL114083 as reference compounds and aim to hit potent inhibitors against ALDH2 from a ligand-based virtual screening of world-approved drugs. A variety of computational approaches, such as the 2D and 3D similarity search, molecular docking, toxicity prediction, molecular dynamics (MD) simulations, and the molecular mechanics Poisson−Boltzmann surface area (MM–PBSA) analysis are applied to evaluate the drug compounds and explore receptor–ligand interactions at a molecular level. This work is valuable for the further design of potent inhibitors against ALDH2 for therapeutic treatments of alcohol addiction.

## 2. Results and Discussion

### 2.1. Virtual Screening of World-Approved Drugs via the 2D/3D Similarity Search 

**Figure 1:**
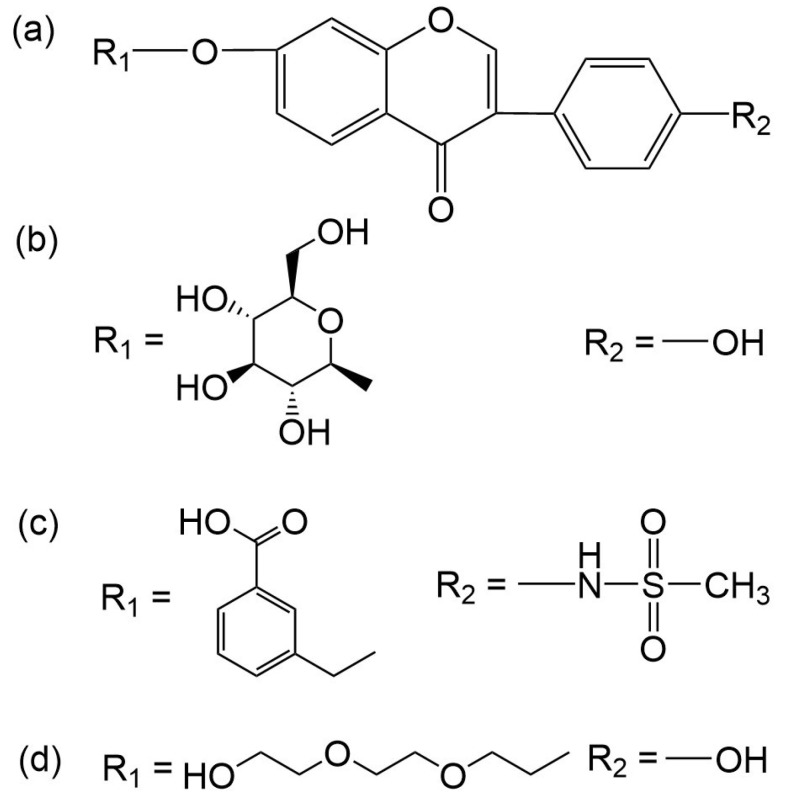
Molecular structures of the reference compounds used for the 2D/3D similarity search. (**a**) Isoflavone skeleton with substituent groups of R_1_ and R_2_ for (**b**) Daidzin (IC_50_ = 0.08 μM), (**c**) CVT-10216 (IC_50_ = 0.029 μM), and (**d**) CHEMBL114083 (IC_50_ = 0.04 μM).

Given the excellent performance of isoflavone analogs, three compounds of Daidzin, CVT-10216, and CHEMBL114083 were chosen as references in the ligand-based 2D/3D similarity search. The molecular structures of the reference compounds are presented in Figure 1, as well as the half-maximal inhibitory concentration (IC_50_) against ALDH2.

The 2D molecular fingerprints decode the 2D structural fragments of chemical molecules using binary bit vectors to calculate and compare the molecular similarity. Due to its high efficiency and effectiveness, it continues to be the top option for preliminary screening when handling large datasets [41]. The Tanimoto coefficient (*T_c_*) is often used as a key indicator for measuring similarity. For two fingerprints, *A* and *B*, *T_c_* is defined as the ratio of their intersection to their union (Equation (1)) [42,43]:

(1) $$T_{c}\left(A,B\right)=\frac{\left|A\cap B\right|}{\left|A\cup B\right|}=\frac{I\left(A,B\right)}{U\left(A,B\right)}$$

where I(A,B) = |A∩B| is the cardinality of the intersection of fingerprints *A* and *B* and represents the number of features present in both fingerprints; U(A,B) = |A∪B| is the union of the two fingerprints and indicates the total number of features present in either *A* or *B*. A higher *T_c_* value means a greater similarity.

We tested four algorithms for generating 2D molecular fingerprints supported in the RDKit toolkit (version 2022.09.5) [44], including MACCS (Molecular ACCess System) Keys [45], RDKit-specific fingerprints, Extended-Connectivity FingerPrints (ECFP4) with a diameter of 4 [46], and Functional-Class FingerPrints FCFP4 (a variant of ECFP) with a diameter of 4. The last two were derived via applying the Morgan algorithm [47] and therefore belonged to a family of Morgan fingerprints (also known as circular fingerprints).

Through the 2D molecular fingerprint similarity search of world-approved drugs (5903 compounds), the top 100 compounds with high *T_c_* values for each combination of the algorithm and the reference compound were collected. For instance, the top 100 *T_c_* values for MACCS keys, RDKit, ECFP4, and FCFP4 fingerprints ranged from 0.649 to 0.974, 0.512 to 0.743, 0.301 to 0.581, and 0.348 to 0.613, respectively, for the search using Daidzin as a reference (Table S1 in the Supplementary Materials). Note that Daidzin is a world-approved drug (ZINC ID: ZINC004098610); its *T_c_* value amounted to 1 and was ignored for a clear presentation of the similarity of other compounds. *T_c_* ranges from 0 to 1, regardless of the fingerprint length, and it greatly depends on the used type of fingerprints [48]. Therefore, a direct comparison of the *T_c_* values from different algorithms/reference combinations appeared to make no sense.

The 2D molecular fingerprinting describes the topological structures, while 3D similarity-based screening takes into account the physical and chemical factors, such as the atomic distance, electrostatic potential, 3D molecular shape, and pharmacological information. The 3D shape of ligands is directly related to the complementarity of the molecular associations between ligands and receptors, and chemical group proximity is a crucial prerequisite for providing a variety of intermolecular interactions. We conducted the 3D similarity-based screening using the algorithms of E3FP [49] and USRCAT [50]. E3FP stands for the Extended 3D FingerPrint, inspired by the commonly used ECFP (2D) fingerprint, and it integrates tightly with the RDKit toolkit [44]. USRCAT is an extension of the ultrafast shape recognition (USR) algorithm [51,52] with CREDO Atom Types [53]. The top 100 *T_c_* values for E3FP using Daidzin as a reference ranged from 0.173 to 0.346 (Table S2 in the Supplementary Materials). USRCAT is a method based on the 3D molecular shape, and its similarity is assessed by a similarity score; the score for the Daidzin reference range from 0.190 to 0.297 (Table S2). The 2D and 3D similarity search results for the reference compounds of CVT-10216 and CHEMBL114083 are given in Tables S3–S6, respectively, in the Supplementary Material. Due to the flexibility and complexity of the 3D structures, the T_c_ values and molecular similarity scores with 3D similarity-based approaches are marginally lower than that with 2D molecular fingerprints [54]. 

In total, we tested six similarity-search algorithms (four for 2D and two for 3D) using three reference compounds. The collected top 100 compounds for 18 algorithms/reference combinations (Tables S1–S6) were merged together, resulting in a subset of 861 compounds from the world-approved drugs. Considering multiple charge states or isoforms at different (pH) conditions, we obtained 1097 compounds for a further assessment via molecular docking and toxicity prediction.

### 2.2. Assessment via Molecular Docking

#### 2.2.1. Comparison of Similarity-Search Methods

**Figure 2:**
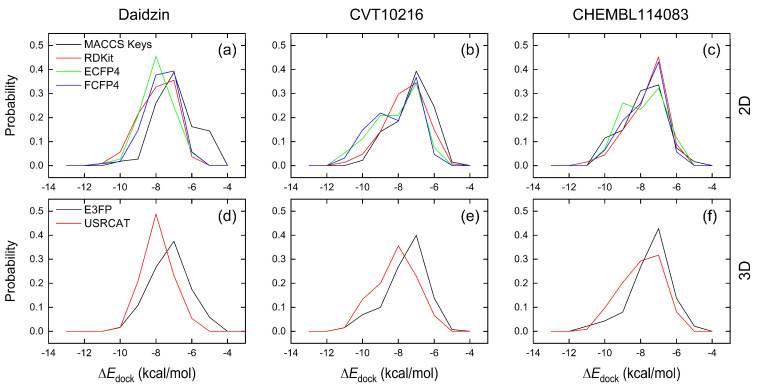
Probability of binding affinities (∆*E*_dock_) from docking predictions between ALDH2 and the top 100 drugs selected from the 2D ((**a**–**c**), top) and 3D ((**d**–**f**), bottom) similarity search using the reference compounds of Daidzin (**left**), CVT-10216 (**middle**), and CHEMBL114083 (**right**).

In order to compare different algorithms for the selection of hit compounds, we calculated the binding affinities (∆*E*_dock_) of the top 100 compounds with the receptor (ALDH2 plus NAD^+^) via molecular docking. In the absence of NAD^+^, the ligand enters into the cofactor-binding domain. For the 2D molecular fingerprint similarity search, ECFP4 appeared to have more of a chance to select a compound with a strong binding strength with ALDH2 than the other methods of MACCS Keys, RDKit, and FCFP4 in most cases, as indicated by a higher probability at more negative ∆*E*_dock_ values (Figure 2). For instance, a higher probability for ∆*E*_dock_ ≤ −8 kcal/mol was observed for ECFP4 compared to the other three methods (Figure 2a). When using CVT-10216 as a reference, a similar performance was observed for the probability profiles of ECFP4 and FCFP4 with ∆*E*_dock_ ≤ −9 kcal/mol (Figure 2b). The MACCS Keys fingerprint showed a bad performance with Daidzin and CVT-10216 as reference molecules (Figure 2a,b), while it appeared to be better than the fingerprinting methods of RDKit and FCFP4 (Figure 2c). The good performance of ECFP4 may be ascribed to the fact that it describes detailed information on the structure of atoms and their neighborhoods, such as the number of direct connections of non-hydrogen atoms, the atomic number, atomic charge, and number of connected hydrogen atoms [46].

As a molecular-shape based approach, USRCAT outperformed the 3D molecular fingerprint E3FP (Figure 2d–f). It is well acknowledged that a configurational match is one of the decisive factors favoring molecular associations between binding partners, like the binding of an antigen to an antibody. This may explain the good performance of USRCAT in finding potent inhibitors from a 3D structural view.

Binding affinities (∆*E*_dock_) with the receptor (ALDH2 plus NAD^+^) amounted to −9.1 ± 0.5, −10.4 ± 0.7, and −8.0 ± 0.1 kcal/mol for the three reference compounds of Daidzin, CVT-10216, and CHEMBL114083, respectively. CVT-10216 showed the strongest binding result with ALDH2, in agreement with the IC_50_ values (Figure 1), whereas the Vina scoring failed to reproduce the relative binding strengths of Daidzin and CHEMBL114083. In this work, we aimed to hit compounds with a binding strength similar to or stronger than that of the reference molecules. We therefore used a cutoff of ∆*E*_dock_ = −10 kcal/mol for a further selection of potential inhibitors.

The 2D/3D similarity search hit 33 compounds (∆*E*_dock_ ≤ −10 kcal/mol) with distinct ZINC IDs from world-approved drugs. We were able to select 4, 24, and 17 drugs with the reference molecules of Daidzin, CVT-10216, and CHEMBL114083, respectively (Table 1). It appeared that when using CVT-10216 as the reference, more compounds with strong binding affinities with ALDH2 could be singled out. ECFP4, FCFP4, and USRCAT helped hit 14, 14, and 13 compounds, respectively, and they performed better than the other methods of MACCS Keys, RDKit, and E3FP, in line with the results stated above (Figure 2). The performances of the 2D molecular fingerprint methods of MACCS Keys and RDKit appeared to not be sensitive to the used references, and they yielded few hits (Table 1).

During our tests, the ECFP4 (2D), FCFP4 (2D), and USRCAT (3D) similarity search methods showed an overall good performance. Note that some compounds could only be hit by one single method, as indicated by the numbers in parenthesis in Table 1. Using CVT-10216 as a reference, for instance, Sorafenib N-Oxide (ZINC003817152) and N-Desmethyl Imatinib (ZINC021981222) could only be selected via ECFP4. Similarly, FCFP4 hit both enantiomers of Sarizotan (*R*-type: ZINC000021067; *S*-type: ZINC000006990) with the reference CVT-10216; the 3D USRCAT method generated six hits (Table 1). In general, it is better to utilize different search algorithms and principles to capture the key and abundant physical and chemical characteristics for obtaining hits in the virtual screening of a large chemical database [34,55,56,57,58].

#### 2.2.2. Molecular Docking Prediction

Considering the different charge states and isomers, we obtained 42 hits in total from the docking predictions with binding affinities (∆*E*_dock_) ≤ −10 kcal/mol (Table 2). Eltrombopag (ZINC011679756, *q* = −3) showed the strongest binding strength with ∆*E*_dock_ = −11.2 kcal/mol. Two more compounds of Indacaterol-8-*O*-Glucuronide (ZINC049783754) and Eltrombopag (*q* = −2) showed a binding of ∆*E*_dock_ ≤ −11 kcal/mol. Note that the compounds were neutral (*q* = 0), if not stated otherwise. Isomer I of pranlukast (-IA and -IB; ZINC001542146) displayed a stronger binding result than its isomer II (-IIA and -IIB; ZINC015919406) by 0.7 kcal/mol. For the different charge states of Eltrombopag (ZINC011679756), Netarsudil (ZINC113149554), Troglitazone (ZINC000968278), and 5-O-Desmethyldonepezil (ZINC013449462), the Vina scoring [59] was able to generate different binding affinities. However, the Vina docking predicted identical binding affinities for the neutral and positively charged (*q* = 1) compounds of 6-*O*-desmethyldonepeil (ZINC013449412) and Sequinavir (ZINC026985532). This finding implies that the Vina scoring failed to predict the relative binding strengths for the compounds with different charge states in some cases. It may be related to an issue of not using atomic charges directly for the evaluation of electrostatic interactions in the Vina scoring, as stated in the previous work [60].

Table 2 also lists the Tanimoto coefficients (for the first five methods based on 2D/3D molecular fingerprints) and similarity scores (for USRCAT) using CVT-10216 as the reference molecule. The values using different references are presented in the Supplementary Materials (Tables S1–S6). The last column in Table 2 indicates the used references that are able to single out the hits. Daidzin helped hit four compounds of ZINC000256630457, ZINC000057674 (Flavone), ZINC049783754 (Indacaterol-8-O-Glucuronide), and 17-Alpha-Estradiol-3-Glucuronide (ZINC013515303), as indicated by “Y/X/X” (X = Y/N) in Table 2; the first one could be selected by only using the USRCAT method (Table 1) with the reference of Daidzin (“Y/N/N” in Table 2). CVT-10216 and CHEMBL114083 helped with the selection of 18 and 8 compounds, as indicated by “N/Y/N” and “N/N/Y”, respectively (Table 2). Detailed information on the hits using different methods or references are given in the Supplementary Materials (Table S7). Of the 42 hits, only Flavone (ZINC000057674) could be selected using all of the three reference molecules, and this could only be performed via the FCFP4 method, as indicated by “Y/Y/Y” in Table 2 and Table S7.

### 2.3. Assessment via a Toxicity Evaluation

The liver serves as the main detoxification organ for drugs and the principal metabolic site upon alcohol intake. Therefore, an essential selection criterion is that potential drugs are expected to have less toxic outcomes and side effects. We assessed the toxicities of the selected 42 potential ALDH2 inhibitors (Table 2) using the ProTox-II online tool [47]. The compounds that were predicted to be non-toxic were marked as N and the toxic compounds were marked as Y; a confidence estimate for the prediction is given in parenthesis (Table 3). Organ toxicity (hepatotoxicity) and four toxicological endpoints (cytotoxicity, mutagenicity, carcinogenicity, and immunotoxicity) were predicted and are presented in Table 3. Hepatotoxicity indicates liver dysfunction or liver damage in association with an overload of drugs or xenobiotics [47]. 

The compounds that were predicated to be toxic with a confidence value lower than 0.6 or nontoxic for all of the tested toxicities were selected for the subsequent analysis. For the FDA-approved drugs, only Sequinavir, Fexofenadine, and Naproxen met such toxicity criteria (Table 3). Fexofenadine (ZINC003824921; denoted as Fexofenadine-I) was already investigated in our previous work [60], and it was used for a comparison with the hits in this work. Based on the toxicity assessment, we selected 15 compounds in total for the MD simulations, including four isomers of Pranlukast (IA, IB, IIA, and IIB; *q* = −1), different charge states of Sequinavir (*q* = 0/+1), Netarsudil (*q* = 0/+1), and Troglitazone (*q* = 0/−1), both enantiomers (*q* = +1) of Sarizotan, Zeaxanthin, Fexofenadine-II (ZINC003872566), and Naproxen (Table 3).

### 2.4. MD Simulation and Binding Energy Calculation

**Figure 3:**
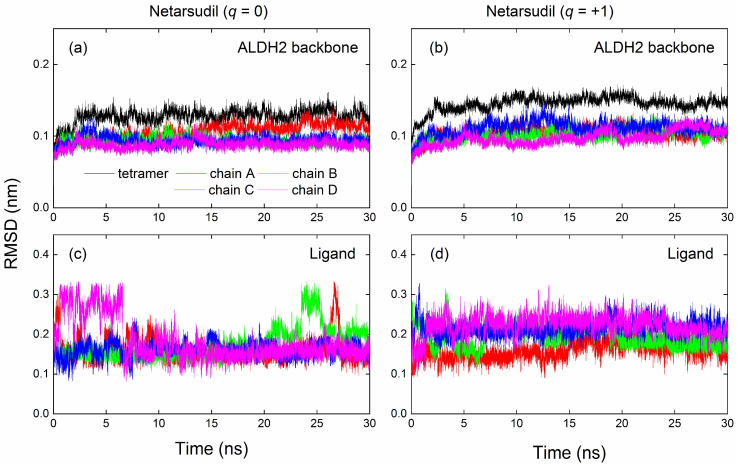
Root-mean-square deviations (RMSDs) of protein backbone atoms (**a**,**b**) of the ALDH2 tetramer and corresponding monomers (chains A–D) from the crystal structure as a function of simulation time for ALDH2 complexes with the neutral (**left**) and charged (**right**) states of netarsudil. The ligand RMSDs from the initial configurations (i.e., docking poses) were calculated excluding hydrogen atoms (**c**,**d**).

We performed 30 ns molecular dynamics (MD) simulations of the ALDH2 tetramer in the presence of the selected inhibitors and cofactor NAD^+^. The root-mean-square deviations (RMSDs) of backbone atoms of the ALDH2 monomers and a tetramer from the crystal structure as a function of the simulation time for the complexes with Netarsudil (*q* = 0/+1) are presented in Figure 3. For the neutral and positively charged states, the RMSDs of the tetramer were well converged and amounted to 0.13 and 0.15 nm, respectively during the MD simulations, revealing that the protein structures were well maintained (Figure 3a,b). Compared to the apo (ligand-free) form of ALDH2, the binding with the positively charged Netarsudil resulted in a greater fluctuation of the overall tetramer structure, while no significant changes in the RMSDs for the corresponding four monomers were observed (Figure 3 and Table 4). The RMSD values for the ALDH2 complexes with the selected 15 inhibitors as well as the three reference molecules are tabulated in Table 4. Although ligand binding may yield a large RMSD value of ca. 0.2 nm in some cases, the ALDH2 tetramers can be well maintained with an RMSD of ≤0.16 nm (Table 4). The RMSDs of Netarsudil (*q* = 0/+1) from their initial configurations (i.e., docking poses) are presented in Figure 3c,d; the values for other compounds are given in Table S8. Similar to the protein backbones, the ligand structures tended to achieve an equilibrium state upon binding with the receptor after 10 ns MD simulations, as indicated by the small standard deviations (0.01–0.05 nm) for the ligand RMSDs in Table S8. In almost all cases, ALDH2 and the ligand structures achieved an overall stable state during the last 10 ns simulations, and the trajectories (i.e., snapshots of receptor–ligand complexes) in this time interval were used for the calculation of binding energies upon the association of receptor and ligand molecules.

After removing water molecules and ions from the simulation trajectories, 100 receptor–ligand frames were subjected to the molecular mechanics Poisson–Boltzmann surface area (MM–PBSA) analysis for the binding energy calculations. The cofactor NAD^+^ was adjacent to the ligand-binding domain of ALDH2, and it was regarded as one of the receptor residues in the calculation. There were differences in the binding energies (∆*E*_bind_) of different monomers (Table 4), and we used the Boltzmann factor as a weight [61,62] to compute the averaged binding energies (<∆*E*_bind_>) over the four monomers (Equation (2)):

(2) $$∆E_{\mathrm{bind}}=\frac{\sum_{i}∆E_{\mathrm{bind},i}\exp\left(-∆E_{\mathrm{bind},i}/RT\right)}{\sum_{i}\exp\left(-∆E_{\mathrm{bind},i}/RT\right)}$$

where *i* indicates the monomer chain (A–D), *R* is the ideal gas constant, and *T* is the temperature (*K*). The monomer with a lower ∆*E*_bind_ had a heavier weight and <Δ*E*_bind_> was therefore very close to the lowest value of the four monomers.

The binding energies (<∆*E*_bind_>) between the receptor (ALDH2 plus NAD^+^) and ligand for the three reference molecules of Daidzin, CVT-10216, and CHEMBL114083 amounted to −26.3, −35.5, and −23.5 kcal/mol, respectively (Table 4). Positively charged compounds yielded binding energies of −65.4 kcal/mol for Sequinavir and ca. −60 kcal/mol for *R*/*S* Sarizotan and Netarsudil. Neutral drugs of Zeaxanthin and Troglitazone presented predictions of −45.9 and −40.3 kcal/mol, respectively, showing a stronger binding strength than CVT-10216. Neutral states of Sequinavir and Netarsudil produced binding energies of −27.5 and −24.5 kcal/mol, respectively, similar to that with Daidzin and CHEMBL114083. The other six compounds with a negative charge (*q* = −1) showed near-zero or positive values for ∆*E*_bind_, implying that the complexation with these ligands were thermodynamically unfavorable.

The binding energy (∆*E*_bind_) can be further divided into four contributions from van der Waals (∆*E*_vdw_), electrostatic (∆*E*_elec_), polar (∆*G*_polar_), and nonpolar (∆*G*_nonpolar_) interactions; the sum of the first two is the MM part (Δ*E*_MM_) and the last two provide the solvation contribution (Δ*G*_sol_). In all cases, ∆*E*_vdw_ and ∆*G*_nonpolar_ favored ligand binding, whereas ∆*G*_polar_ showed the opposite (Table 5). Due to the great contribution of ∆*G*_polar_, the solvation part (Δ*G*_sol_) did not favor the binding; this was probably related to the (unfavorable) desolvation of receptor and ligand molecules upon complexation [61,62,63]. The receptor had a negative net charge (*q* = −6 for ALDH2), and the unfavorable binding with negatively charged inhibitors (mentioned above) may have been due to the electrostatic repulsion of the like sign between the binding partners, as indicated by the positive ∆*E*_elec_ (Table 5). On the contrary, the positively charged compounds (*q* = +1) contributed considerably (∆*E*_elec_ < −70 kcal/mol) due to the electrostatic attraction, giving rise to the strongest binding outcome with ALDH2 (Table 4 and Table 5).

Based on the MM–PBSA analysis, we selected five potential inhibitors against ALDH2, namely, Sequinavir (*q* = +1), Sarizotan (*R*/*S*; *q* = +1), Netarsudil (*q* = 0/+1), Zeaxanthin (*q* = 0), and Troglitazone (*q* = 0). The first one was approved by the FDA; however, it was not singled out in our previously docking-based screening [60]. In that work, we hit Fexofenadine (ZINC003824921) with Δ*E*_bind_ = −22.5 kcal/mol [60], showing a weaker binding strength than the selected five compounds in this work (Table 5). Its isomer (Fexofenadine-II, ZINC003872566) was not approved by the FDA, and it yielded an almost identical prediction (−21.7 kcal/mol, Table 5).

### 2.5. Identification of the Key Residues for Ligand Binding

**Figure 4:**
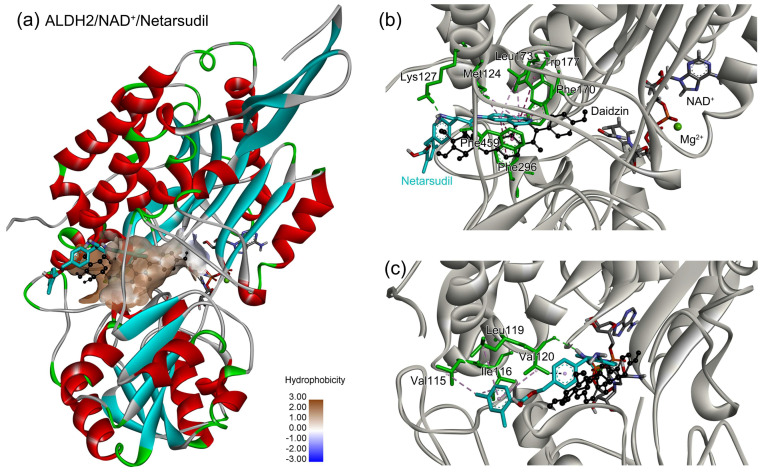
Representative binding poses of ALDH2 with Netarsudil (*q* = 0) during MD simulations. The receptor ALDH2 is displayed as a solid ribbon model; Netarsudil is shown as a stick model and its carbon atoms are colored in cyan. Daidzin is represented by the ball and stick model (in black) to depict the hydrophobic tunnel for ligand binding in the crystal structure of protein 2VLE (**a**). A comparison with Daidzin indicates the relative position of Netarsudil in the ligand-binding tunnel. The cofactor NAD^+^ is shown with the ball and stick model and is colored by element, and the Mg^2+^ ion is represented by a green ball. Panel (**b**) presents the interactions between Netarsudil and protein residues (in green) inside the ligand-binding tunnel, and panel (**c**) shows the interactions with the residues located in the entrance or outside of the tunnel. Receptor−ligand interactions are indicated by dashed lines.

**Figure 5:**
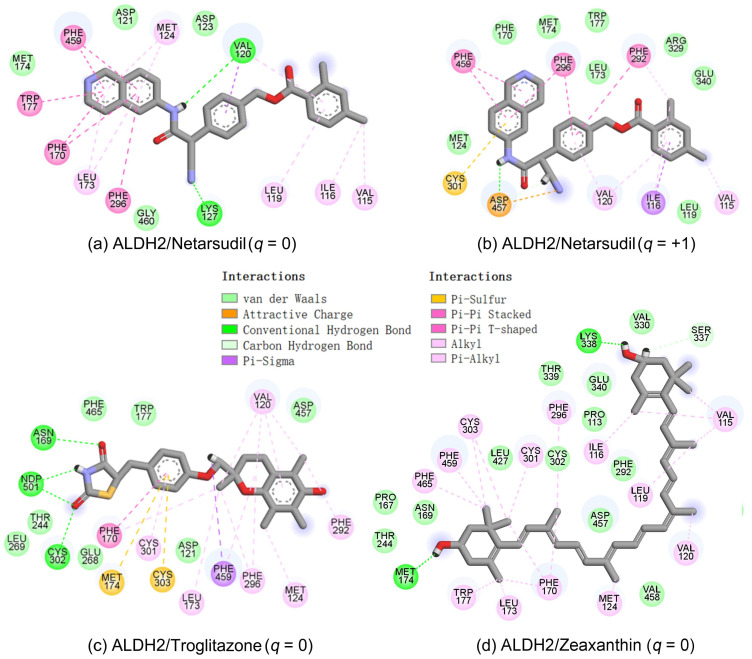
Two-dimensional diagrams of receptor–ligand interactions for the ALDH2 complexes with (**a**) Netarsudil (*q* = 0), (**b**) Netarsudil (*q* = +1), (**c**) Troglitazone (*q* = 0), and (**d**) Zeaxanthin (*q* = 0). C, H, O, N, and S atoms of the ligands are colored in gray, white, red, blue, and orange, respectively. The cofactor NAD^+^ is regarded as one of the receptor residues (NDP501). Averaged snapshots from the last 10 ns MD simulations were used to generate the diagrams, and the interaction types are presented by different colors. Left moieties of the inhibitors were inserted into the hydrophobic ligand-binding tunnel of ALDH2.

After the assessments via a similarity search, molecular docking, toxicity prediction, and MM–PBSA analysis, the five compounds of Sequinavir (*q* = +1), Sarizotan (*R*/*S*; *q* = +1), Netarsudil (*q* = 0/+1), Zeaxanthin (*q* = 0), and Troglitazone (*q* = 0) were hit and used to investigate receptor–ligand interactions at a molecular level. Compared with the crystal ALDH2/Daidzin complex, Netarsudil was not allowed to penetrate deep into the hydrophobic ligand-binding tunnel (Figure 4a), and its strong binding with ALDH2 was attributed to the interactions with protein residues both inside the tunnel (Figure 4b) and in the entrance or outside of the tunnel (Figure 4c). Inside the tunnel, protein residues with aromatic rings, such as Phe170, Trp177, Phe296, and Phe459, offered π–π interactions, and Met124 and Leu173 provided π–alkyl interactions (Figure 4b and Figure 5a). In the entrance or outside of the tunnel, Val120 and Lys127 formed hydrogen bonds with Netarsudil (*q* = 0), Val115 and Ile116 offered alkyl interactions, and Leu119 and Val120 provided π–alkyl interactions (Figure 4c and Figure 5a). The positively charged Netarsudil also showed a shallow penetration into the ligand-binding tunnel, whereas the binding pose was different from its neutral state (Figure 5a,b). The catalytic site of ALDH2 was located at the bottom of the ligand-binding tunnel and was adjacent to the cofactor-binding domain. Considering the shallow penetration, Netarsudil was likely not an ideal inhibitor against ALDH2 because it did not fully occupy the ligand-binding tunnel, leaving a space for substrate binding (Figure 4). A similar shallow penetration was observed for both enantiomers of Sarizotan.

**Figure 6:**
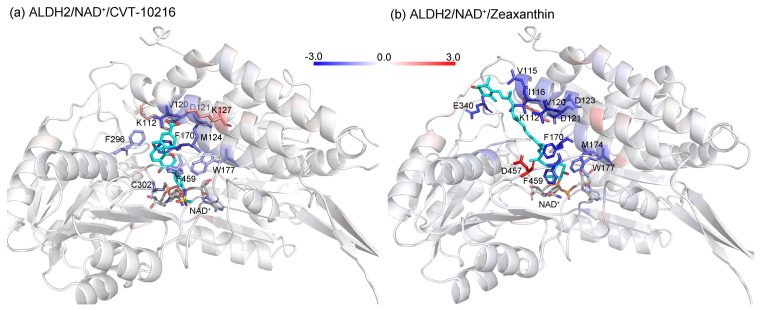
The mapping of residue-wise energy contributions (kcal/mol) on the ALDH2 structures for binding with CVT-10216 (**a**) and Zeaxanthin (**b**). Carbon atoms of the ligands are colored in cyan. Key residues with a contribution of ≥1 kcal/mol are shown with a stick model and colored by their energy contributions; the blue resides indicate the stabilization of the ligand, while the red ones disfavor the binding. The PDB files containing energy values in the B factor were obtained via the “energy2bfac” module in the “g_mmpbsa” package [64], and were used to generate this figure by PyMOL (https://www.pymol.org (accessed on 30 June 2023); version 2.5.4).

Unlike Netarsudil, Sequinavir, Troglitazone, and Zeaxanthin penetrated deep into the ligand-binding tunnel. For instance, the cofactor NAD^+^ (residue NDP501) and the inhibitor Troglitazone formed hydrogen bonds (Figure 5c). Glu268, which was located at the bottom of the hydrophobic tunnel, formed van der Waals contacts with Troglitazone (Figure 5c). Another residue, Asn169, at the bottom of the tunnel, provided hydrogen bonding and van der Waals interactions with Troglitazone (Figure 5c) and Zeaxanthin (Figure 5d), respectively. Phe170 and Phe296 were located in the middle of the hydrophobic tunnel and they offered π–π and π–alkyl interactions. Sulfur-containing residues, such as Met174 and Cys303, provided π–sulfur interactions with Troglitazone, while Met174 displayed van der Waals interactions with Netarsudil. Ile116 and Val120 were situated at the entrance of the ligand-binding tunnel and they were able to offer a variety of interactions, such as alkyl, π–alkyl, and hydrogen bonding with the inhibitors (Figure 5). Compared with CVT-10216 (Figure 6a), Zeaxanthin interacted with more protein residues, such as Val115, Ile116, Lys338, and E340 (Figure 5d and Figure 6b).

**Figure 7:**
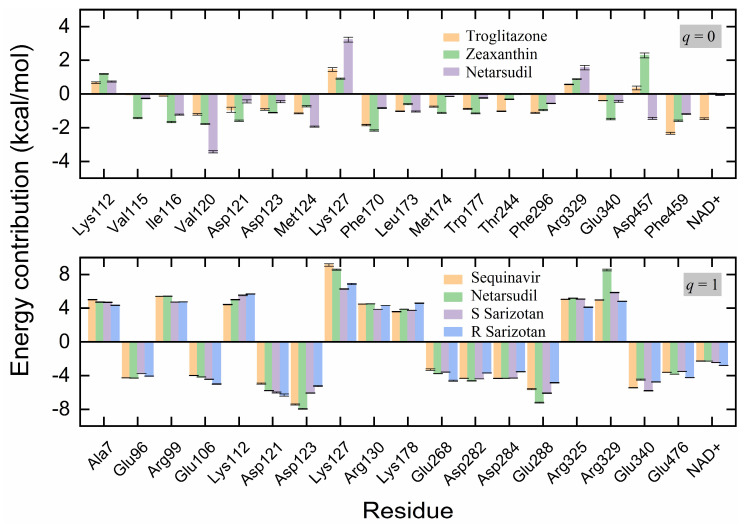
Energy contributions (kcal/mol) per residue to the binding of the receptor ALDH2 with the selected neutral (**top**) and positively charged (**bottom**) inhibitors of Zeaxanthin (*q* = 0), Troglitazone (*q* = 0), Netarsudil (*q* = 0/+1), Sequinavir (*q* = +1), and *R*/*S Sarizotan* (*q* = +1). The shown residues contribute ≥ 1 kcal/mol (**top**) or 4 kcal/mol (**bottom**) to the binding with at least one of the inhibitors.

The receptor−ligand-binding energies were further decomposed into the energy contributions per residue for identifying the key residues responsible for the binding. The residues with a contribution of ≥1 kcal/mol for at least one of the neutral inhibitors are presented in Figure 7. In most cases, the protein residues and cofactor NAD^+^ that were located at the bottom, in the middle, or at the entrance of the ligand-binding tunnel favored the ligand-binding process. However, the charged residues, such as Lys112, Lys127, Arg329, and Asp457, appeared to disfavor the binding for all of the neutral inhibitors, except for Asp457, with a favorable contribution to the binding with Netarsudil (Figure 7, top). The disfavor was mainly due to the considerable contributions from polar interactions (i.e., positive Δ*G*_polar_ values), as shown in Table S9 in the Supplementary Materials. For instance, Asp457 formed van der Waals contacts with Zeaxanthin (Figure 5d and Figure 6b) with a favorable Δ*E*_MM_ of −1.45 kcal/mol, while Δ*G*_polar_ and Δ*G*_nonpolar_ amounted to 3.93 and −0.20 kcal/mol, respectively, resulting in a total Δ*E*_bind_ of 2.28 kcal/mol (Figure 7 and Table S9).

For binding with positively charged compounds (*q* = +1), the key residues were almost the amino acids with a net charge, except for Ala7 (Figure 7, bottom). Ala7 at the N-terminal of ALDH2 was close to the entrance of the ligand-binding tunnel and it disfavored the binding of inhibitors. Because of the electrostatic repulsion, positively charged residues, such as Lys and Arg, disfavored the binding. On the contrary, negatively charged residues, such as Asp and Glu, displayed favorable contributions due to the electrostatic attraction. The cofactor NAD^+^ still favored the binding of the inhibitors to the receptor ALDH2. For these residues, the main contributions were from Δ*E*_MM_, while the solvation (Δ*G*_sol_) did not contribute much to the binding in most cases (Tables S10 and S11).

## 3. Computational Methods

### 3.1. Ligand-Based Similarity Search

#### 3.1.1. Reference Molecule and Drug Database for the Similarity Search

Molecules with similar chemical structures and/or three-dimensional shapes might have comparable physiological functions and activities and, hence, it would yield a good match with, for instance, the binding site of the target protein (i.e., receptor). Following this principle, we chose three isoflavone analogs of Daidzin, CVT-10216, and CHEMBL114083 (https://www.ebi.ac.uk/chembl; accessed on 30 June 2023) as the reference molecules (Figure 1) for the ligand-based similarity search, in order to hit potential drug molecules for the inhibition against ALDH2. 

The world-approved drugs were extracted from the ZINC [65,66] online database (https://zinc.docking.org/substances/subsets/world; accessed on 30 June 2023). As of the accessed date, there were 5903 approved drugs in major jurisdictions, including the U.S. Food and Drug Administration (FDA). The 3D structures of some drugs were available in the ZINC database and they were given at different pH conditions (Reference: pH = 7.4; Middle: pH = 6.4–8.4; Low: pH = 5.4–6.4; High: pH = 8.4–9.4) [65]. If not available, we calculated the structures at pH = 7.4 (i.e., the Reference condition) via the Open Babel toolbox (version 3.1.0) [67]. Considering multiple charge states or isoforms at different (pH) conditions, we obtained a set of 7658 drug compounds for use in total, and Daidzin belonged to this set (ZINC ID: ZINC004098610).

#### 3.1.2. The 2D/3D Similarity Search

The open-source RDKit toolkit (version 2022.09.5) [44] was used to generate molecular fingerprints and calculate molecular similarities. Four build-in methods for generating two-dimensional (2D) fingerprints were tested, namely, RDKit-specific (topological) fingerprints, MACCS Keys [45], Extended-Connectivity FingerPrints (ECFP4) [46], and Functional-Class FingerPrints FCFP4 (a variant of ECFP). The last two were derived via applying the Morgan algorithm [47] and therefore belonged to a family of Morgan fingerprints (also known as circular fingerprints). The SMILES (simplified molecular input line entry system) format of 5903 drug compounds from the ZINC database [65,66] was converted to the MOL format via the RDKit toolkit [44], and then used for obtaining 2D molecular fingerprints.

Three-dimensional (3D) similarity searches were conducted using the algorithms of E3FP [49] and USRCAT [50]. E3FP stands for the Extended 3D FingerPrint, inspired by the commonly used ECFP (2D) fingerprint, and it tightly integrates with the RDKit toolkit (https://anaconda.org/conda-forge/e3fp; accessed on 30 June 2023). USRCAT is an extension of the ultrafast shape recognition (USR) algorithm [51,52] with CREDO Atom Types [53], and it is a built-in module in the RDKit toolkit. The SMILES format of the drug compounds at pH = 7.4 was generated via the Open Babel toolbox (version 3.1.0) [67] and then converted to 3D conformations in the SDF format via the Biovia Discovery studio visualizer software (version 2019). The SDF files were used to generate 3D fingerprints (for E3FPs) or perform a molecular-shape-based similarity evaluation (for USRCAT).

The similarity of the 2D/3D molecular fingerprints was evaluated via a metric of Tanimoto coefficients (*Tc*), and for the USRCAT method, a metric of similarity scores was used. Both metrics ranged from 0 to 1, and a higher value indicated a higher similarity. In total, we tested six methods for the 2D/3D similarity screening of the world-approved drugs, using three reference compounds (Section 3.1.1). The top 100 hits for each search were merged for a further evaluation.

### 3.2. Docking Protocol

#### 3.2.1. Ligand and Receptor Preparations

The molecular structures of the hit compounds from the similarity search were taken from the ZINC 15 database [65,66] in the MOL2 format; if not available, the 3D structures were generated from the SMILES files via the Biovia Discovery studio visualizer software (version 2019). A python script (prepare_ligand4.py) in the MGLTools package (version 1.5.6, https://ccsb.scripps.edu/mgltools; accessed on 30 June 2023) [68] was used to prepare the ligand PDBQT files for the docking calculation in batch mode.

The crystal structure of the receptor ALDH2 were retrieved from the Protein Data Bank (PDB) database (PDB code: 2VLE); it formed a complex with Daidzin and the cofactor NAD^+^ was not yet determined [31]. Following the previous work [60,69], we constructed a tetramer of ALDH2/Daidzin/NAD^+^ complexes, and NAD^+^ was regarded as one of the receptor residues in the docking. The AutoDockTools module in the MGLTools package (version 1.5.6) [68] was used to generate the receptor PDBQT file via adding polar hydrogens, computing Gasteiger charges, and assigning AD4 atom types.

#### 3.2.2. Docking Calculation

Autodock Vina software (version 1.1.2) [59] was used to conduct docking predictions in the batch mode. The search space was 3 × 3 × 3 nm^3^ with center values of x = 9.14, y = 1.65, and z = 7.08 nm, roughly close to the geometrical center of Daidzin. Default values were used for other parameters in the Vina docking. Such a protocol was validated in our previous work, where the docking pose of Daidzin agreed well with its crystal state [69]. The docking was implemented 10 times with random seeds for each ligand, and the binding poses with the strongest binding affinities were used for the data collection. Note that the Vina docking did not use explicit atomic charges for scoring [59], although it used the PDBQT files as inputs. The atomic charges in the PDBQT files were therefore ignored in the Vina scoring.

### 3.3. Toxicity Prediction

The hit compounds after two rounds of screening (i.e., similarly search and docking prediction) were submitted to the ProTox-II online server [70] to predict the toxicity (https://tox-new.charite.de/protox_II, accessed on 30 June 2023). Toxicological endpoints of mutagenicity, carcinogenicity, cytotoxicity, and immunotoxicity were tested as well as the organ toxicity (hepatotoxicity).

### 3.4. Molecular Simulation Protocol

The selected drug inhibitors and reference compounds were subjected to 30 ns molecular dynamics (MD) simulations in the tetramer forms of ALDH2/ligand/NAD^+^ complexes in physiological salt concentrations of 0.15 mol/L. For instance, the length of the simulation cell for CVT-10216 was ca. 10.5 nm, containing an ALDH2 tetrameter, 4 ligands (CVT-10216), 4 cofactors (NAD^+^), 4 Mg^2+^ ions, 123 Na^+^, 103 Cl^−^, and 27,758 water molecules. We performed production MD simulations at the NPT ensemble (P = 1 bar; T = 298.15 *K*) using GROMACS software (version 2018.4) [71], with a time step of 0.002 ps. For a comparison with the previous work [60,69], the Amber 99SB-ILDN force field [72] was used to model ALDH2 and the General Amber Force Field (GAFF) [73] was used for the ligands. Force-field parameters of NAD^+^ [74,75] were obtained from a collection by the group of Richard Bryce (http://research.bmh.manchester.ac.uk/bryce/amber; accessed on 30 June 2023). The rigid TIP3P model [76] was used to describe water molecules. Restricted electrostatic potential (RESP) charges were assigned to the ligands. The calculation of the RESP charges and more details on the simulation protocol were presented in our previous work [60,69].

### 3.5. MM–PBSA Analysis

The molecular mechanics Poisson−Boltzmann surface area (MM–PBSA) analysis has the advantage of evaluating the driving forces quantitatively and identifying the key residues responsible for molecular associations. Here, we used the “g_mmpbsa” toolkit [64] to perform such an analysis using the last 10 ns simulation trajectories. Mg^2+^, Na^+^, and Cl^−^ ions and water molecules were removed, and the atoms that jumped across the simulation box were put back. This ensured that the receptor/ligand molecules remained whole, and this could be performed using the GROMACS tool of “gmx trjconv” with an option of “-pbc nojump” [71]. The trajectories were saved with an interval of 100 ps and we obtained 100 snapshots in total for use in the MM–PBSA analysis.

The binding free energy (∆*G*_bind_) of molecular associations, in general, consists of molecular mechanics (MM, ∆*E*_MM_), solvation (∆*G*_sol_), and entropy (∆*S*) contributions, as given in Equation (3):

∆*G*_bind_ = ∆*E*_MM_ + ∆*G*_sol_ – *T*∆S = ∆*E*_vdW_ + ∆*E*_elec_ + ∆*G*_polar_ + ∆*G*_nonpolar_ – *T*∆S (3)

∆*E*_MM_ is a sum of van der Waals (∆*E*_vdW_) and electrostatic (∆*E*_elec_) interactions, and ∆*G*_sol_ can be decomposed into polar (∆*G*_polar_) and nonpolar (∆*G*_nonpolar_) solvation contributions. The entropy calculation has yet not been supported by the “g_mmpbsa” toolkit [64], and we therefore did not consider the entropy contribution; the calculated values are known as the binding energy (∆*E*_bind_). Δ*G*_polar_ was calculated using the built-in APBS package [77], and for Δ*G*_nonpolar_, the solvent accessible surface area (SASA) model was used for the calculation. Python scripts of “MmPbSaStat.py” and “MmPbSaDecomp.py”, written by the developer team of “g_mmpbsa” (http://rashmikumari.github.io/g_mmpbsa/Usage.htm; accessed on 30 June 2023), were used to address the data statistics and energy decomposition. The Biovia Discovery Studio visualization software (version 2019) was used to generate 2D/3D figures to depict the receptor−ligand interactions and the key residues identified by the MM–PBSA analysis.

## 4. Conclusions

In this work, a variety of computational approaches, such as the 2D/3D similarity search, molecular docking, toxicity prediction, molecular dynamics (MD) simulation, and the molecular mechanics Poisson−Boltzmann surface area (MM–PBSA) analysis were used to perform a ligand-based virtual screening for repurposing world-approved drugs as potential inhibitors against ALDH2. Using the reported inhibitors of Daidzin, CVT-10216, and CHEMBL114083 as reference compounds, we hit two neutral drugs of Zeaxanthin and Troglitazone with almost no toxicity, and the binding energies (∆*E*_bind_) with ALDH2 for these two compounds were −45.9 and −40.3 kcal/mol, respectively. Interestingly, both compounds could be hit using CVT-10216 and CHEMBL114083 as references, but it failed for Daidzin. The binding strength of both compounds appeared stronger than the previously reported potent inhibitor of CVT-10216 (IC_50_ = 0.029 μM [18]; ∆*E*_bind_ = −35.5 kcal/mol). Sequinavir (*q* = +1) yielded a binding value of -65.4 kcal/mol and it was promising as well. Although each enantiomer of Sarizotan (*q* = +1) and different charge states of Netarsudil (*q* = 0/+1) also showed a strong binding outcome with ALDH2, they showed a shallow penetration into the substrate-binding hydrophobic tunnel of ALDH2 and could not fully occupy it. Therefore, they might not be ideal candidates for the inhibition against ALDH2.

Sequinavir was already approved by the FDA (a subset of world-approved drugs), while it was not singled out in our previous study on the docking-based screening of FDA-approved drugs [60]. In that work, we hit two compounds of Butenafine (*q* = +1; ∆*E*_bind_ = −74.9 kcal/mol) and Olaparib (*q* = 0; ∆*E*_bind_ = −30.5 kcal/mol), and Fexofenadine (ZINC003824921) was also hit with Δ*E*_bind_ = −22.5 kcal/mol [60]. In this work, we hit Fexofenadine and its isomer (ZINC003872566, not yet approved by the FDA); the isomer produced an almost identical Δ*E*_bind_ of −21.7 kcal/mol. Interestingly, Butenafine and Olaparib were not singled out from the ligand-based similarity search. That is, the similarity of these two compounds with the used reference molecules was very low. This implied a limitation to using the 2D/3D similarity search as a preliminary screening method before the docking evaluation.

The net charge of inhibitors was of great importance for the binding with ALDH2 (*q* = −6 *e*). The positively charged compounds gave rise to a strong binding outcome, whereas the negatively charged ones corresponded to a very weak binding result (positive ∆*E*_bind_). This was likely ascribed to electrostatic attraction and repulsion. In our case, unfortunately, such electrostatic interactions were not accurately considered by the 2D/3D similarity search and the Vina scoring, as indicated by the positive values of ∆*E*_bind_ from the MM–PBSA analysis of the negatively charged drugs. The ligand-based similarity search indeed helped us discover more potent hits, and a good performance was observed for the 2D molecular-fingerprint-based methods of ECFP4 and FCFP4, as well as the 3D molecular-shape-based algorithm of USRCAT. Surprisingly, the 3D molecular-fingerprint-based algorithm of E3FP showed a worse performance than the 2D molecular fingerprints of ECFP4 and FCFP4. This finding reveals the importance of 3D structural matches in the field of drug discovery.

## Figures and Tables

**Table 1:** The number of hit compounds with a strong binding strength (∆*E*_dock_ ≤ −10 kcal/mol) using different methods and reference molecules.

Reference	2D				3D		Total
	MACCS Keys	RDKit	ECFP4	FCFP4	E3FP	USRCAT	
Daidzin	0 (0)	2 (0)	1 (0)	2 (0)	0 (0)	1 (1)	4 (1)
CVT-10216	1 (0)	4 (0)	11 (2)	11 (2)	4 (1)	11 (4)	24 (9)
CHEMBL114083	4 (1)	3 (0)	3 (0)	4 (0)	3 (0)	7 (1)	17 (2)
Total	5 (1)	6 (0)	14 (2)	14 (2)	5 (1)	13 (6)	33 (12)

Some compounds can be hit using more than one method or reference. The values in parenthesis are the numbers of compounds that can only be hit using the corresponding method(s)/reference(s).

**Table 2:** Selected compounds (binding affinities ∆*E*_dock_ ≤ −10 kcal/mol) from the world-approved drug database via the 2D/3D similarity search.

ZINC ID	Molecular Structure	Name	*q*	∆*E*_dock_	MACCS Keys	RDKit	ECFP4	FCFP4	E3FP	USRCAT	Hit
ZINC011679756	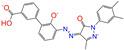	Eltrombopag	−3	−11.2	0.456	0.371	0.285	0.370	0.114	0.097	N/Y/N
ZINC049783754	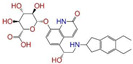	Indacaterol-8-*O*-Glucuronide	0	−11.2	0.451	0.379	0.190	0.240	0.132	0.078	Y/N/Y
ZINC011679756	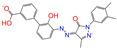	Eltrombopag	−2	−11.0	0.456	0.371	0.285	0.370	0.114	0.097	N/Y/N
ZINC001542146	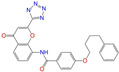	Pranlukast-IA	−1	−10.9	0.494	0.424	0.318	0.411	0.153	0.104	N/Y/Y
ZINC001542146	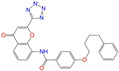	Pranlukast-IB	−1	−10.9	0.494	0.424	0.318	0.411	0.153	0.104	N/Y/Y
ZINC095618662	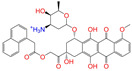	ZINC095618662	1	−10.8	0.527	0.404	0.208	0.266	0.109	0.134	N/Y/N
ZINC019632618	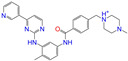	Imatinib-I	1	−10.8	0.292	0.318	0.256	0.322	0.104	0.083	N/Y/N
ZINC019632618	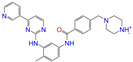	Imatinib-II	1	−10.7	0.292	0.318	0.256	0.322	0.104	0.083	N/Y/N
ZINC003824921	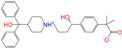	Fexofenadine-I	0	−10.7	0.312	0.235	0.186	0.253	0.082	0.137	N/Y/Y
ZINC021981222	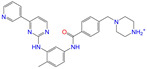	*N*-Desmethyl Imatinib	1	−10.7	0.287	0.317	0.259	0.327	0.098	0.092	N/Y/N
ZINC150339055	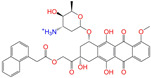	ZINC150339055	1	−10.6	0.527	0.404	0.208	0.266	0.115	0.135	N/Y/N
ZINC008220175	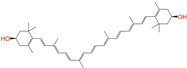	Zeaxanthin	0	−10.6	0.197	0.132	0.035	0.020	0.064	0.114	N/N/Y
ZINC077313075	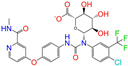	Sorafenib Beta-D-Glucuronide	−1	−10.5	0.469	0.385	0.238	0.323	0.091	0.124	N/Y/N
ZINC113149554	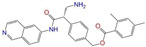	Netarsudil	0	−10.5	0.425	0.298	0.284	0.397	0.127	0.125	N/Y/Y
ZINC001493878	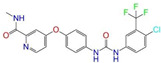	Sorafenib	0	−10.5	0.427	0.298	0.264	0.348	0.092	0.089	N/Y/N
ZINC000968278	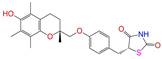	Troglitazone	0	−10.4	0.481	0.311	0.193	0.234	0.089	0.172	N/Y/Y
ZINC000968278	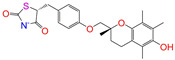	Troglitazone	−1	−10.3	0.481	0.311	0.193	0.234	0.089	0.172	N/Y/Y
ZINC013449462	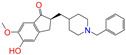	5-*O*-Desmethyldonepezil-I	0	−10.3	0.403	0.279	0.188	0.213	0.097	0.108	N/N/Y
ZINC003872566	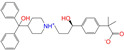	Fexofenadine-II	0	−10.3	0.312	0.235	0.186	0.253	0.079	0.142	N/Y/Y
ZINC000057674	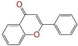	Flavone	0	−10.3	0.322	0.427	0.233	0.375	0.096	0.062	Y/Y/Y
ZINC000021067	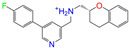	*R* Sarizotan	1	−10.3	0.256	0.305	0.223	0.360	0.058	0.069	N/Y/N
ZINC006037085	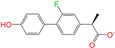	(*R*)-4′-Hydroxyflurbipron	−1	−10.3	0.312	0.247	0.250	0.295	0.078	0.077	N/N/Y
ZINC068202099	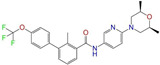	Erismodegib	0	−10.3	0.393	0.349	0.291	0.394	0.106	0.131	N/Y/Y
ZINC003817152	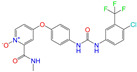	Sorafenib *N*-Oxide	0	−10.3	0.469	0.309	0.260	0.324	0.104	0.087	N/Y/N
ZINC000896717	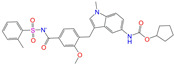	Accolate	−1	−10.2	0.639	0.368	0.290	0.357	0.122	0.103	N/Y/N
ZINC001550477	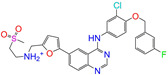	Lapatinib	1	−10.2	0.551	0.410	0.359	0.387	0.160	0.083	N/Y/N
ZINC013515303	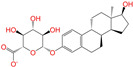	17-Alpha-Estradiol-3-Glucuronide	−1	−10.2	0.368	0.341	0.110	0.159	0.103	0.095	Y/N/Y
ZINC015919406	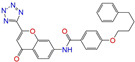	Pranlukast-IIA	−1	−10.2	0.494	0.434	0.354	0.432	0.174	0.099	N/Y/Y
ZINC013449412	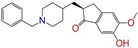	6-*O*-Desmethyldonepeil	0	−10.1	0.403	0.277	0.188	0.213	0.118	0.093	N/N/Y
ZINC013449412	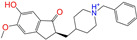	6-*O*-Desmethyldonepeil	1	−10.1	0.403	0.277	0.188	0.213	0.118	0.093	N/N/Y
ZINC006030312	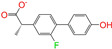	(*S*)-4′-Hydroxyflurbipron	−1	−10.1	0.312	0.247	0.250	0.295	0.075	0.077	N/N/Y
ZINC113149554	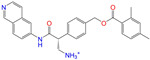	Netarsudil	1	−10.1	0.425	0.298	0.284	0.397	0.127	0.125	N/Y/Y
ZINC015919406	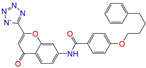	Pranlukast-IIB	−1	−10.1	0.494	0.434	0.354	0.432	0.174	0.099	N/Y/Y
ZINC013449462	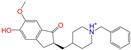	5-*O*-Desmethyldonepezil-I	1	−10.1	0.403	0.279	0.188	0.213	0.097	0.108	N/N/Y
ZINC013449465	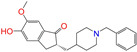	5-*O*-Desmethyldonepezil-II	0	−10.0	0.403	0.279	0.188	0.213	0.111	0.101	N/N/Y
ZINC000006990	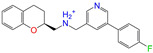	*S* Sarizotan	1	−10.0	0.256	0.305	0.223	0.360	0.074	0.089	N/Y/N
ZINC000105216	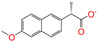	Naproxen	−1	−10.0	0.328	0.204	0.197	0.308	0.161	0.062	N/Y/N
ZINC256630457	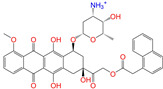	ZINC256630457	1	−10.0	0.527	0.404	0.208	0.266	0.118	0.101	Y/N/N
ZINC256630463	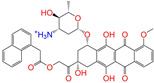	ZINC256630463	1	−10.0	0.527	0.404	0.208	0.266	0.119	0.131	N/Y/N
ZINC028639340	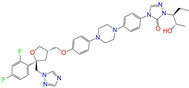	Posaconazole	0	−10.0	0.426	0.393	0.194	0.263	0.075	0.125	N/Y/Y
ZINC026985532	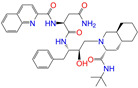	Sequinavir	0	−10.0	0.323	0.349	0.173	0.255	0.095	0.121	N/Y/N
ZINC026985532	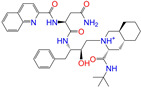	Sequinavir	1	−10.0	0.323	0.349	0.173	0.255	0.095	0.121	N/Y/N

ZINC ID is the compound ID in the ZINC database, *q* is the net charge (*e*), and ∆*E*_dock_ is the binding affinity with ALDH2 from the docking predictions. The Tanimoto coefficients for the molecular fingerprint similarity search using the 2D methods of MACCS Keys, RDKit, ECFP4, and FCFP4 and the 3D method of E3FP were given for the selected compounds. The column “USRCAT” lists the 3D similarity scores using the USRCAT approach. The values for the Tanimoto coefficients and similarity scores correspond to the drugs at the reference pH and are calculated using the CVT-10216 as the reference compound. The last column indicates the reference compounds (Daidzin/CVT-10216/CHEMBL114083) used to hit drugs. For instance, N/Y/Y means this compound can be hit using CVT-10216 or CHEMBL114083 as the reference compounds (Y, short for yes), but it cannot be hit using Daidzin as the reference (N, short for no). The results for the 2D/3D similarity search using different reference compounds are presented in the Supplementary Materials (Tables S1–S6). Pranlukast (*q* = −1 *e*) has four isomers that are named Pranlukast-IA, Pranlukast-IB, Pranlukast-IIA, and Pranlukast-IIB, so do Imatinib, Fexofenadine, and 5-O-Desmethyldonepezil.

**Table 3:** Toxicity predictions for the 42 selected compounds in Table 2 via the ProTox-II platform.

ZINC ID	Name	*q*	Toxicity					FDA
			Dill	Carcino	Immuno	Mutagen	Cyto	
ZINC011679756	Eltrombopag	−3	Y (0.67)	N (0.57)	N (0.72)	N (0.56)	N (0.84)	yes
ZINC049783754	Indacaterol-8-*O*-Glucuronide	0	N (0.71)	N (0.61)	Y (0.87)	N (0.59)	N (0.54)	no
ZINC011679756	Eltrombopag	−2	Y (0.67)	N (0.57)	N (0.72)	N (0.56)	N (0.84)	yes
ZINC001542146	Pranlukast-IA	−1	Y (0.57)	N (0.72)	N (0.87)	Y (0.53)	N (0.77)	no
ZINC001542146	Pranlukast-IB	−1	Y (0.57)	N (0.72)	N (0.87)	Y (0.53)	N (0.77)	no
ZINC095618662	ZINC095618662	1	N (0.85)	N (0.85)	Y (0.99)	Y (0.94)	Y (0.79)	no
ZINC019632618	Imatinib-I	1	Y (0.71)	N (0.67)	Y (0.66)	N (0.73)	N (0.52)	yes
ZINC019632618	Imatinib-II	1	Y (0.71)	N (0.67)	Y (0.66)	N (0.73)	N (0.52)	yes
ZINC003824921	Fexofenadine-I	0	N (0.99)	Y (0.50)	N (0.86)	N (0.85)	N (0.81)	yes
ZINC021981222	*N*-Desmethyl Imatinib	1	N (0.61)	N (0.62)	Y (0.66)	N (0.69)	N (0.60)	no
ZINC150339055	ZINC150339055	1	N (0.85)	N (0.85)	Y (0.99)	Y (0.94)	Y (0.79)	no
ZINC008220175	Zeaxanthin	0	N (0.79)	N (0.67)	N (0.92)	N (0.81)	N (0.89)	no
ZINC077313075	Sorafenib Beta-D-Glucuronide	−1	Y (0.65)	N (0.60)	Y (0.92)	N (0.74)	N (0.63)	no
ZINC113149554	Netarsudil	0	N (0.72)	N (0.52)	N (0.95)	N (0.58)	N (0.59)	no
ZINC001493878	Sorafenib	0	Y (0.82)	N (0.50)	Y (0.92)	N (0.79)	Y (0.77)	yes
ZINC000968278	Troglitazone	0	N (0.62)	N (0.62)	N (0.90)	N (0.58)	N (0.61)	no
ZINC000968278	Troglitazone	−1	N (0.62)	N (0.62)	N (0.90)	N (0.58)	N (0.61)	no
ZINC013449462	5-*O*-Desmethyldonepezil-I	0	N (0.97)	N (0.55)	Y (0.98)	N (0.55)	Y (0.58)	no
ZINC003872566	Fexofenadin-II	0	N (0.99)	Y (0.50)	N (0.86)	N (0.85)	N (0.81)	yes
ZINC000057674	Flavone	0	N (0.70)	Y (0.69)	N (0.99)	N (0.54)	Y (0.75)	no
ZINC000021067	*R* Sarizotan	1	N (0.71)	N (0.62)	N (0.87)	N (0.62)	N (0.62)	no
ZINC006037085	(*R*)-4′-Hydroxyflurbipron	−1	Y (0.68)	N (0.66)	N (0.99)	N (0.85)	N (0.54)	no
ZINC068202099	Erismodegib	0	N (0.52)	N (0.60)	Y (0.85)	N (0.67)	N (0.69)	yes
ZINC003817152	Sorafenib N-Oxide	0	Y (0.67)	N (0.58)	Y (0.76)	Y (0.54)	Y (0.54)	no
ZINC000896717	Accolate	−1	Y (0.76)	N (0.57)	N (0.65)	N (0.67)	N (0.56)	yes
ZINC001550477	Lapatinib	1	Y (0.80)	N (0.55)	Y (0.96)	N (0.51)	Y (0.76)	yes
ZINC013515303	17-Alpha-Estradiol-3-Glucuronide	−1	N (0.84)	N (0.70)	Y (0.99)	N (0.78)	N (0.58)	no
ZINC015919406	Pranlukast-IIA	−1	Y (0.57)	N (0.72)	N (0.87)	Y (0.53)	N (0.77)	no
ZINC013449412	6-*O*-Desmethyldonepeil	0	N (0.98)	N (0.54)	Y (0.98)	N (0.54)	Y (0.65)	no
ZINC013449412	6-*O*-Desmethyldonepeil	1	N (0.98)	N (0.54)	Y (0.98)	N (0.54)	Y (0.65)	no
ZINC006030312	(*S*)-4′-Hydroxyflurbipron	−1	Y (0.68)	N (0.66)	N (0.99)	N (0.85)	N (0.54)	no
ZINC113149554	Netarsudil	1	N (0.72)	N (0.52)	N (0.95)	N (0.58)	N (0.59)	no
ZINC015919406	Pranlukast-IIB	−1	Y (0.57)	N (0.72)	N (0.87)	Y (0.53)	N (0.77)	no
ZINC013449462	5-*O*-Desmethyldonepezil-I	1	N (0.97)	N (0.55)	Y (0.98)	N (0.55)	Y (0.58)	no
ZINC013449465	5-*O*-Desmethyldonepezil-II	0	N (0.97)	N (0.55)	Y (0.98)	N (0.55)	Y (0.58)	no
ZINC000006990	*S* Sarizotan	1	N (0.71)	N (0.62)	N (0.87)	N (0.62)	N (0.62)	no
ZINC000105216	Naproxen	−1	Y (0.51)	N (0.53)	N (0.85)	N (0.74)	N (0.80)	yes
ZINC256630457	ZINC256630457	1	N (0.85)	N (0.85)	Y (0.99)	Y (0.94)	Y (0.79)	no
ZINC256630463	ZINC256630463	1	N (0.85)	N (0.85)	Y (0.99)	Y (0.94)	Y (0.79)	no
ZINC028639340	Posaconazole	0	Y (0.86)	N (0.62)	Y (0.99)	N (0.56)	N (0.75)	yes
ZINC026985532	Sequinavir	0	N (0.60)	N (0.63)	N (0.97)	N (0.79)	N (0.80)	yes
ZINC026985532	Sequinavir	1	N (0.60)	N (0.63)	N (0.97)	N (0.79)	N (0.80)	yes

ZINC ID is the compound ID in the ZINC database. Toxicity predictions include hepatotoxicity (dili for short), carcinogenicity (carcino), immunotoxicity (immune), mutagenicity (mutagen), and cytotoxicity (cyto); N indicates inactive and Y indicates active. The numbers given in parenthesis are the confidence values for the predictions. The last column indicates whether the compound has been approved by the U.S. Food and Drug Administration (FDA) or not.

**Table 4:** Root-mean-square deviations (RMSDs) of protein backbones of the ALDH2 tetramer and the corresponding monomers (chains A–D) from the crystal structure and the binding energies (∆*E*_bind_) between the receptor (ALDH2 plus NAD^+^) and selected inhibitors.

**Name**	***q***	**Chain A**	**Chain B**	**Chain C**	**Chain D**	**Tetramer**
RMSD (nm)						
Sequinavir	1	0.15	0.13	0.14	0.13	0.15
*R* Sarizotan	1	0.17	0.12	0.13	0.13	0.15
*S* Sarizotan	1	0.18	0.12	0.13	0.16	0.17
Netarsudil	1	0.10	0.11	0.11	0.11	0.15
Zeaxanthin	0	0.14	0.11	0.13	0.13	0.14
Troglitazone	0	0.11	0.11	0.11	0.09	0.15
Sequinavir	0	0.19	0.11	0.13	0.12	0.15
Netarsudil	0	0.11	0.09	0.09	0.09	0.13
Fexofenadine-II	0	0.17	0.12	0.13	0.12	0.14
Troglitazone	−1	0.14	0.13	0.13	0.13	0.15
Pranlukast-IA	−1	0.14	0.10	0.13	0.17	0.15
Pranlukast-IIB	−1	0.20	0.11	0.15	0.15	0.16
Pranlukast-IIA	−1	0.19	0.12	0.13	0.12	0.16
Pranlukast-IB	−1	0.14	0.13	0.13	0.13	0.14
Naproxen	−1	0.14	0.12	0.14	0.18	0.16
Daidzin	0	0.09	0.11	0.10	0.09	0.12
CVT-10216	0	0.18	0.11	0.16	0.13	0.16
CHEMBL114083	0	0.17	0.12	0.14	0.15	0.16
ligand-free		0.10	0.10	0.12	0.11	0.13
**Name**	***q***	**Chain A**	**Chain B**	**Chain C**	**Chain D**	**<∆*E*_bind_>**
∆*E*_bind_ (kcal/mol)						
Sequinavir	1	−56.6 ± 2.4	−61.1 ± 1.2	−65.4 ± 0.5	−43.1 ± 1.7	−65.4 ± 0.5
*R* Sarizotan	1	−54.5 ± 1.5	−56.9 ± 0.6	−60.8 ± 0.3	−55.8 ± 1.6	−60.8 ± 0.3
*S* Sarizotan	1	−59.3 ± 1.8	−55.6 ± 0.6	−57.4 ± 0.7	−60.3 ± 1.2	−60.1 ± 1.3
Netarsudil	1	−52.7 ± 2.3	−59.7 ± 1.1	−57.3 ± 0.9	−57.1 ± 2.7	−59.6 ± 1.6
Zeaxanthin	0	−45.9 ± 1.3	−42.4 ± 0.8	−34.1 ± 1.7	−38.2 ± 0.8	−45.9 ± 1.3
Troglitazone	0	−40.3 ± 1.8	−25.5 ± 1.0	−28.2 ± 1.3	−26.3 ± 1.5	−40.3 ± 1.8
Sequinavir	0	−22.0 ± 2.0	−27.5 ± 1.4	−18.4 ± 1.6	−14.1 ± 2.4	−27.5 ± 1.4
Netarsudil	0	−21.3 ± 2.4	−21.4 ± 1.5	−24.6 ± 0.9	−19.8 ± 0.4	−24.5 ± 0.6
Fexofenadine-II	0	−19.8 ± 4.1	−9.2 ± 1.1	−21.7 ± 1.5	−5.7 ± 1.3	−21.7 ± 1.7
Troglitazone	−1	−0.2 ± 2.0	12.9 ± 1.1	12.0 ± 1.3	16.8 ± 1.8	−0.2 ± 2.0
Pranlukast-IA	−1	14.4 ± 0.8	3.1 ± 1.6	0.7 ± 0.2	16.6 ± 0.7	0.7 ± 0.2
Pranlukast-IIB	−1	9.7 ± 1.5	18.9 ± 2.2	11.2 ± 6.0	5.5 ± 3.6	5.5 ± 1.3
Pranlukast-IIA	−1	12.4 ± 2.2	12.6 ± 0.9	18.4 ± 2.9	17.6 ± 2.8	12.5 ± 0.8
Pranlukast-IB	−1	20.2 ± 1.2	16.3 ± 1.4	19.4 ± 2.7	15.6 ± 1.5	15.7 ± 1.7
Naproxen	−1	22.1 ± 0.3	22.9 ± 0.7	28.9 ± 3.5	16.3 ± 1.0	16.3 ± 1.0
Daidzin	0	−19.0 ± 2.4	−22.0 ± 1.1	−26.3 ± 1.1	−20.8 ± 1.2	−26.3 ± 1.5
CVT-10216	0	−27.3 ± 1.7	−25.1 ± 1.2	−35.5 ± 1.1	−29.3 ± 1.1	−35.5 ± 1.1
CHEMBL114083	0	−23.5 ± 0.5	−22.1 ± 1.1	−23.6 ± 1.1	−12.9 ± 1.5	−23.5 ± 0.5

The selected inhibitors are taken from Table 3 with low toxicities. *q* is the net charge (*e*) of the inhibitors. The last 10 ns simulation trajectories were used to compute the RMSDs and ∆*E*_bind_. <∆E_bind_> is the averaged binding energy over the monomers (chains A–D) and is weighted by their Boltzmann factors. Standard deviations for RMSDs are less than 0.01 nm and are not presented here. Block averaging was used for the binding energy calculations for obtaining good statistics. The data for Daidzin and the ligand-free form of ALDH2 were taken from Ref. [60].

**Table 5:** Decomposition of the binding energies (kcal/mol) of the selected inhibitors with ALDH2 using the MM–PBSA analysis of the last 10 ns simulation trajectories.

Name	FDA	*q*	pH	Δ*E*_vdW_	Δ*E*_elec_	Δ*E*_MM_	Δ*G*_polar_	Δ*G*_nonpolar_	Δ*G*_sol_	Δ*E*_bind_
Sequinavir	yes	1	ref	−50.9 ± 1.1	−70.3 ± 1.2	−121.2 ± 0.8	61.2 ± 0.5	−5.3 ± 0.1	55.9 ± 0.5	−65.4 ± 0.5
*R* Sarizotan	no	1	ref	−40.5 ± 0.4	−89.1 ± 1.6	−129.6 ± 1.7	73.4 ± 1.7	−4.6 ± 0.0	68.8 ± 1.7	−60.8 ± 0.3
*S* Sarizotan	no	1	ref	−38.1 ± 0.9	−84.6 ± 1.7	−122.7 ± 1.6	66.8 ± 2.2	−4.4 ± 0.0	62.4 ± 2.1	−60.3 ± 1.2
Netarsudil	no	1	ref	−47.6 ± 0.4	−88.5 ± 1.3	−136.1 ± 1.5	81.7 ± 1.5	−5.3 ± 0.1	76.4 ± 1.5	−59.7 ± 1.1
Zeaxanthin	no	0	ref	−65.7 ± 2.1	−3.9 ± 0.7	−69.7 ± 2.2	31.0 ± 1.4	−7.2 ± 0.1	23.8 ± 1.4	−45.9 ± 1.3
Troglitazone	no	0	lo	−57.2 ± 0.9	−7.1 ± 0.5	−64.3 ± 0.9	29.3 ± 1.2	−5.3 ± 0.1	24.0 ± 1.2	−40.3 ± 1.8
Sequinavir	yes	0	hi	−48.6 ± 2.7	−10.8 ± 2.2	−59.4 ± 4.8	37.0 ± 4.4	−5.2 ± 0.3	31.9 ± 4.1	−27.5 ± 1.4
Netarsudil	no	0	hi	−42.6 ± 1.1	−12.3 ± 1.4	−54.9 ± 1.7	35.1 ± 1.6	−4.8 ± 0.0	30.3 ± 1.5	−24.6 ± 0.9
Fexofenadine-II	yes	0	ref	−45.2 ± 0.8	−59.4 ± 1.2	−104.6 ± 1.3	88.0 ± 2.3	−5.2 ± 0.1	82.9 ± 2.3	−21.7 ± 1.5
Troglitazone	no	−1	ref	−57.5 ± 1.0	8.8 ± 0.6	−48.7 ± 1.6	53.6 ± 0.7	−5.2 ± 0.0	48.4 ± 0.7	−0.2 ± 2.0
Pranlukast-IA	no	−1	mid	−66.4 ± 0.7	19.0 ± 1.2	−47.4 ± 1.7	54.1 ± 1.6	−6.1 ± 0.1	48.1 ± 1.6	0.7 ± 0.2
Pranlukast-IIB	no	−1	mid	−50.5 ± 3.1	19.6 ± 4.2	−30.9 ± 7.0	41.2 ± 6.0	−4.8 ± 0.2	36.4 ± 5.8	5.5 ± 3.6
Pranlukast-IIA	no	−1	ref	−48.4 ± 1.6	44.6 ± 1.6	−3.9 ± 0.2	21.4 ± 1.0	−4.9 ± 0.1	16.5 ± 0.9	12.6 ± 0.9
Pranlukast-IB	no	−1	ref	−47.7 ± 1.4	53.7 ± 4.0	6.0 ± 4.7	14.6 ± 3.7	−5.0 ± 0.1	9.6 ± 3.7	15.6 ± 1.5
Naproxen	yes	−1	ref	−32.8 ± 0.5	9.5 ± 1.4	−23.2 ± 1.6	42.8 ± 1.2	−3.3 ± 0.0	39.5 ± 1.2	16.3 ± 1.0
Daidzin	no	0		−53.3 ± 0.9	−15.6 ± 0.6	−68.9 ± 0.8	47.3 ± 0.9	−4.7 ± 0.0	42.5 ± 0.9	−26.3 ± 1.1
CVT-10216	no	0		−62.3 ± 0.8	−23.2 ± 0.6	−85.5 ± 1.0	55.6 ± 0.8	−5.6 ± 0.0	50.0 ± 0.8	−35.5 ± 1.1
CHEMBL114083	no	0		−44.8 ± 1.3	−7.8 ± 1.1	−52.6 ± 1.2	33.7 ± 1.8	−4.6 ± 0.1	29.1 ± 1.7	−23.5 ± 0.5

The decomposition energies are for the monomers with the lowest binding energies (Table 3). Column 2 indicates whether the compound was approved by the FDA or not. *q* is the net charge (*e*) of the compounds and *q* = −6 *e* for the ALDH2 monomer. Different pH values include the Reference (ref, pH = 7.4), Middle (mid, 6.4–8.4), Low (lo, 5.4–6.4), and High (hi, 8.4–9.4) conditions. ∆*E*_MM_ is the MM part and is the sum of ∆*E*_vdW_ and ∆*E*_elec_. ∆*G*_sol_ is the solvation part and amounts to ∆*G*_polar_ plus ∆*G*_nonpolar_. The binding energies for the three reference compounds are also presented at the bottom; the energies for Daidzin were taken from Ref. [60].

## Data Availability

Data is contained within the article.

## Supplementary Materials

The following supporting information can be downloaded at: https://www.mdpi.com/article/10.3390/molecules28217325/s1; Tables S1–S6: top 100 compounds from the 2D/3D similarity search using different reference compounds; Table S7: detailed information on hits; Table S8: ligand RMSDs; Tables S9–S11: energy decomposition for identified key residues.
