# Supplementary material for: Repurposing Drugs for Inhibition against ALDH2 via a 2D/3D Ligand-Based Similarity Search and Molecular Simulation
Source: Molecules. 2023 Oct 29;28(21):7325. doi: 10.3390/molecules28217325 (PMC10650273; doi:10.3390/molecules28217325)
Supplement: Supplementary file 1 [file molecules-28-07325-s001.zip › molecules-2634301-supplementary.pdf]

Supplementary Materials for:

Repurposing Drugs for Inhibition against ALDH2 via a 2D/3D Ligand-Based Similarity Search and Molecular Simulation

Wanyun Jiang, Junzhao Chen, Puyu Zhang, Nannan Zheng, Le Ma, Yongguang Zhang and Haiyang Zhang*

Department of Biological Science and Engineering, School of Chemistry and Biological Engineering, University of Science and Technology Beijing, 100083 Beijing, China

*Correspondence: zhanghy@ustb.edu.cn

Table S1. Selected drugs from the 2D similarity search using daidzin as a reference compound.

ZINC ID	MACCS keys Similarity	RDkit Similarity	ECFP Similarity	FCFP Similarity
ZINC003860441	0.974	0.662	0.485	0.613
ZINC072206342	0.950	0.648	0.349	0.478
ZINC004096846	0.950	0.648	0.349	0.478
ZINC003977786	0.925	0.424	0.397	0.432
ZINC003977787	0.925	0.424	0.397	0.432
ZINC003977803	0.905	0.743	0.365	0.496
ZINC004098512	0.905	0.743	0.365	0.496
ZINC003978654	0.902	0.433	0.339	0.327
ZINC095862725	0.886	0.646	0.310	0.416
ZINC085552699	0.886	0.646	0.310	0.416
ZINC004098633	0.825	0.532	0.528	0.573
ZINC000518554	0.821	0.547	0.581	0.600
ZINC000058117	0.795	0.305	0.214	0.291
ZINC034114798	0.780	0.694	0.376	0.564
ZINC064622163	0.780	0.321	0.225	0.217
ZINC004095761	0.775	0.314	0.272	0.272
ZINC085479227	0.775	0.314	0.178	0.178
ZINC008217411	0.775	0.314	0.215	0.215
ZINC085552114	0.775	0.314	0.178	0.178
ZINC012493465	0.775	0.314	0.272	0.272
ZINC085551979	0.775	0.312	0.178	0.178
ZINC299818012	0.775	0.313	0.132	0.132
ZINC004095762	0.775	0.314	0.272	0.272
ZINC004095793	0.775	0.314	0.272	0.272
ZINC006920404	0.775	0.314	0.215	0.215
ZINC095619107	0.756	0.313	0.284	0.284
ZINC000039092	0.756	0.308	0.208	0.295
ZINC095619106	0.756	0.313	0.284	0.284
ZINC033902810	0.750	0.321	0.287	0.287
ZINC000119983	0.750	0.304	0.224	0.315
ZINC100054755	0.745	0.450	0.205	0.274
ZINC100054746	0.745	0.450	0.205	0.274
ZINC100054749	0.745	0.450	0.205	0.274
ZINC095618817	0.745	0.450	0.205	0.274
ZINC003869685	0.744	0.478	0.252	0.371
ZINC002539702	0.744	0.239	0.250	0.292
ZINC018185774	0.744	0.474	0.246	0.379
ZINC003819138	0.739	0.375	0.409	0.434
ZINC004777909	0.729	0.353	0.181	0.232

ZINC039163942	0.729	0.353	0.181	0.232
ZINC004782900	0.729	0.353	0.181	0.232
ZINC001668755	0.729	0.353	0.181	0.232
ZINC003938684	0.729	0.450	0.194	0.261
ZINC068197809	0.725	0.435	0.282	0.377
ZINC003872446	0.725	0.348	0.161	0.252
ZINC003869855	0.725	0.342	0.230	0.303
ZINC261494652	0.720	0.495	0.199	0.257
ZINC261494650	0.720	0.495	0.199	0.257
ZINC261494651	0.720	0.495	0.199	0.257
ZINC261494653	0.720	0.495	0.199	0.257
ZINC003861213	0.718	0.298	0.292	0.292
ZINC003833800	0.718	0.298	0.292	0.292
ZINC004099009	0.714	0.451	0.207	0.301
ZINC004099008	0.714	0.451	0.207	0.301
ZINC100051419	0.714	0.423	0.228	0.373
ZINC005225520	0.707	0.313	0.263	0.263
ZINC005733652	0.707	0.491	0.239	0.408
ZINC095618751	0.707	0.389	0.227	0.339
ZINC036520252	0.700	0.395	0.398	0.410
ZINC001530604	0.698	0.455	0.209	0.316
ZINC001530788	0.698	0.449	0.185	0.297
ZINC001530605	0.698	0.455	0.209	0.316
ZINC013831818	0.692	0.151	0.168	0.194
ZINC100051402	0.690	0.408	0.313	0.411
ZINC100051398	0.690	0.415	0.269	0.385
ZINC005159763	0.690	0.432	0.291	0.398
ZINC100051405	0.690	0.415	0.269	0.385
ZINC100051400	0.690	0.408	0.313	0.411
ZINC005599377	0.683	0.379	0.304	0.377
ZINC033986666	0.683	0.387	0.270	0.352
ZINC033986664	0.683	0.383	0.281	0.364
ZINC100053131	0.683	0.342	0.306	0.355
ZINC004217475	0.682	0.335	0.272	0.272
ZINC261494704	0.681	0.356	0.155	0.150
ZINC261494702	0.681	0.356	0.155	0.150
ZINC261494701	0.681	0.356	0.155	0.150
ZINC245224178	0.681	0.356	0.136	0.131
ZINC261494703	0.681	0.356	0.155	0.150
ZINC084589076	0.667	0.386	0.211	0.342
ZINC003977952	0.667	0.328	0.272	0.272
ZINC100006264	0.667	0.386	0.211	0.342
ZINC043207238	0.660	0.394	0.413	0.437
ZINC003874715	0.660	0.367	0.158	0.216

ZINC003861806	0.660	0.367	0.158	0.216
ZINC095618769	0.659	0.425	0.304	0.370
ZINC006030359	0.659	0.425	0.304	0.370
ZINC100051411	0.659	0.422	0.228	0.385
ZINC100051415	0.659	0.422	0.228	0.385
ZINC013829478	0.659	0.530	0.277	0.336
ZINC004329335	0.651	0.308	0.250	0.250
ZINC001529215	0.650	0.202	0.146	0.209
ZINC001529214	0.650	0.202	0.146	0.209
ZINC261494676	0.649	0.439	0.210	0.301
ZINC100054198	0.649	0.439	0.210	0.301
ZINC261494675	0.649	0.439	0.210	0.301
ZINC100054205	0.649	0.439	0.210	0.301
ZINC100054201	0.649	0.439	0.210	0.301
ZINC261494674	0.649	0.439	0.210	0.301
ZINC261494673	0.649	0.439	0.210	0.301
ZINC011616153	0.492	0.546	0.223	0.336
ZINC011616152	0.492	0.546	0.223	0.336
ZINC011616154	0.492	0.546	0.223	0.336
ZINC014879999	0.492	0.546	0.223	0.336
ZINC003831231	0.492	0.546	0.223	0.336
ZINC100053654	0.552	0.540	0.305	0.340
ZINC100053656	0.552	0.540	0.305	0.340
ZINC100053657	0.552	0.540	0.305	0.340
ZINC261494625	0.552	0.540	0.305	0.340
ZINC261494626	0.552	0.540	0.305	0.340
ZINC261494624	0.552	0.540	0.305	0.340
ZINC100053651	0.552	0.540	0.305	0.340
ZINC261494623	0.552	0.540	0.305	0.340
ZINC077312651	0.614	0.535	0.261	0.304
ZINC005197198	0.510	0.534	0.327	0.352
ZINC062227230	0.574	0.524	0.331	0.331
ZINC253612045	0.574	0.524	0.331	0.331
ZINC253612041	0.574	0.524	0.331	0.331
ZINC100054267	0.574	0.524	0.331	0.331
ZINC062227227	0.574	0.524	0.331	0.331
ZINC100054264	0.574	0.524	0.331	0.331
ZINC150338771	0.558	0.523	0.259	0.304
ZINC150338767	0.558	0.523	0.259	0.304
ZINC033903112	0.646	0.523	0.315	0.364
ZINC077291459	0.615	0.522	0.302	0.323
ZINC077291461	0.615	0.522	0.302	0.323
ZINC000001342	0.644	0.520	0.254	0.443
ZINC095618869	0.492	0.519	0.301	0.336

ZINC095618868	0.492	0.519	0.301	0.336
ZINC040165222	0.608	0.519	0.313	0.373
ZINC004096817	0.615	0.519	0.272	0.291
ZINC013515302	0.615	0.519	0.272	0.291
ZINC256310200	0.615	0.519	0.272	0.291
ZINC008551674	0.615	0.519	0.272	0.291
ZINC012496781	0.615	0.519	0.272	0.291
ZINC150374443	0.615	0.519	0.272	0.291
ZINC013515304	0.615	0.519	0.272	0.291
ZINC013515303	0.615	0.519	0.272	0.291
ZINC261494563	0.563	0.518	0.258	0.287
ZINC095618660	0.563	0.518	0.258	0.287
ZINC100054093	0.563	0.518	0.258	0.287
ZINC100054096	0.563	0.518	0.258	0.287
ZINC095618661	0.563	0.518	0.258	0.287
ZINC261494562	0.563	0.518	0.258	0.287
ZINC004245665	0.597	0.518	0.336	0.425
ZINC004099002	0.593	0.518	0.211	0.254
ZINC000895390	0.538	0.518	0.327	0.457
ZINC003872928	0.524	0.518	0.263	0.455
ZINC261494575	0.500	0.518	0.320	0.331
ZINC095618679	0.500	0.518	0.320	0.331
ZINC100054171	0.500	0.518	0.320	0.331
ZINC095618680	0.500	0.518	0.320	0.331
ZINC100054168	0.500	0.518	0.320	0.331
ZINC261494576	0.500	0.518	0.320	0.331
ZINC022060640	0.567	0.517	0.314	0.417
ZINC102136963	0.615	0.517	0.277	0.296
ZINC008234405	0.615	0.517	0.277	0.296
ZINC102136971	0.615	0.517	0.277	0.296
ZINC040165221	0.615	0.517	0.277	0.296
ZINC040165220	0.615	0.517	0.277	0.296
ZINC040165219	0.615	0.517	0.277	0.296
ZINC100054877	0.484	0.517	0.270	0.290
ZINC095618593	0.596	0.516	0.228	0.219
ZINC100053693	0.596	0.516	0.228	0.219
ZINC261494564	0.596	0.516	0.228	0.219
ZINC095618592	0.596	0.516	0.228	0.219
ZINC100053696	0.596	0.516	0.228	0.219
ZINC261494565	0.596	0.516	0.228	0.219
ZINC077291483	0.561	0.516	0.295	0.315
ZINC077291480	0.561	0.516	0.295	0.315
ZINC049783754	0.547	0.514	0.303	0.366
ZINC095618900	0.547	0.514	0.303	0.366

ZINC261494637	0.556	0.513	0.366	0.356
ZINC100053680	0.556	0.513	0.366	0.356
ZINC100053678	0.556	0.513	0.366	0.356
ZINC261494636	0.556	0.513	0.366	0.356
ZINC095618587	0.556	0.513	0.366	0.356
ZINC095618588	0.556	0.513	0.366	0.356
ZINC095618878	0.366	0.513	0.365	0.406
ZINC095618879	0.366	0.513	0.365	0.406
ZINC253613193	0.532	0.512	0.287	0.317
ZINC253613192	0.532	0.512	0.287	0.317
ZINC100054307	0.532	0.512	0.287	0.317
ZINC100054315	0.532	0.512	0.287	0.317
ZINC100054312	0.532	0.512	0.287	0.317
ZINC100054305	0.532	0.512	0.287	0.317
ZINC253613194	0.532	0.512	0.287	0.317
ZINC253613191	0.532	0.512	0.287	0.317
ZINC031425360	0.475	0.506	0.362	0.373
ZINC031425358	0.475	0.506	0.362	0.373
ZINC031425361	0.475	0.506	0.362	0.373
ZINC031425359	0.475	0.506	0.362	0.373
ZINC077293633	0.522	0.512	0.341	0.362
ZINC077300530	0.515	0.510	0.336	0.356
ZINC095618866	0.516	0.421	0.331	0.397
ZINC095618867	0.516	0.421	0.331	0.397
ZINC084386263	0.524	0.470	0.331	0.370
ZINC031460595	0.500	0.421	0.331	0.311
ZINC031460590	0.500	0.421	0.331	0.311
ZINC253613240	0.447	0.501	0.329	0.348
ZINC100054332	0.447	0.501	0.329	0.348
ZINC253613241	0.447	0.501	0.329	0.348
ZINC100054334	0.447	0.501	0.329	0.348
ZINC253613243	0.447	0.501	0.329	0.348
ZINC253613242	0.447	0.501	0.329	0.348
ZINC261494610	0.552	0.487	0.328	0.328
ZINC100054977	0.552	0.487	0.328	0.328
ZINC100054975	0.552	0.487	0.328	0.328
ZINC261494611	0.552	0.487	0.328	0.328
ZINC095618768	0.609	0.416	0.328	0.383
ZINC095618767	0.609	0.416	0.328	0.383
ZINC031290887	0.517	0.427	0.326	0.326
ZINC031290884	0.517	0.427	0.326	0.326
ZINC003871576	0.641	0.472	0.321	0.414
ZINC261494691	0.461	0.498	0.310	0.348
ZINC100054321	0.461	0.498	0.310	0.348

ZINC261494692	0.461	0.498	0.310	0.348
ZINC261494689	0.461	0.498	0.310	0.348
ZINC100054319	0.461	0.498	0.310	0.348
ZINC261494690	0.461	0.498	0.310	0.348
ZINC002570857	0.371	0.258	0.306	0.270
ZINC004099104	0.522	0.490	0.305	0.378
ZINC077301904	0.402	0.432	0.303	0.338
ZINC095618749	0.479	0.456	0.301	0.333
ZINC095618748	0.479	0.456	0.301	0.333
ZINC095617672	0.475	0.386	0.252	0.408
ZINC095617674	0.483	0.390	0.243	0.397
ZINC001534965	0.519	0.370	0.213	0.379
ZINC000538275	0.524	0.395	0.271	0.376
ZINC003872687	0.554	0.290	0.215	0.374
ZINC000607910	0.554	0.290	0.215	0.374
ZINC033971074	0.382	0.506	0.258	0.372
ZINC095618600	0.382	0.506	0.258	0.372
ZINC003872582	0.634	0.462	0.270	0.372
ZINC077320103	0.414	0.428	0.260	0.368
ZINC077320107	0.414	0.428	0.260	0.368
ZINC001544683	0.492	0.360	0.235	0.363
ZINC022060265	0.483	0.405	0.264	0.362
ZINC095618922	0.508	0.435	0.299	0.359
ZINC095618919	0.508	0.435	0.299	0.359
ZINC095618920	0.508	0.435	0.299	0.359
ZINC095618921	0.508	0.435	0.299	0.359
ZINC001886617	0.433	0.373	0.201	0.358
ZINC001530639	0.433	0.373	0.201	0.358
ZINC095617673	0.491	0.337	0.212	0.356
ZINC000057674	0.487	0.397	0.218	0.354
ZINC000607872	0.544	0.316	0.187	0.352
ZINC002014976	0.544	0.316	0.187	0.352
ZINC095618646	0.508	0.349	0.223	0.352
ZINC095618645	0.508	0.349	0.223	0.352
ZINC001319967	0.385	0.206	0.227	0.352
ZINC003201958	0.385	0.206	0.227	0.352
ZINC000000407	0.385	0.206	0.227	0.352
ZINC011616526	0.385	0.206	0.227	0.352
ZINC009302317	0.385	0.206	0.227	0.352
ZINC003830970	0.385	0.206	0.227	0.352
ZINC022060259	0.483	0.407	0.254	0.350

The top 100 hits via each method are merged together and listed here.

Table S2. Selected drugs from the 3D similarity search using daidzin as a reference compound.

E3FP Similarity		USRCAT Score	
ZINC ID	Similarity	ZINC ID	Score
ZINC003860441	0.346	ZINC004098512	0.297
ZINC004098633	0.310	ZINC004096846	0.269
ZINC003977787	0.268	ZINC261494648	0.259
ZINC003977786	0.253	ZINC085552699	0.257
ZINC004245665	0.250	ZINC150338771	0.254
ZINC003833800	0.246	ZINC022060640	0.253
ZINC072206342	0.23	ZINC031425358	0.247
ZINC000518554	0.228	ZINC001895505	0.242
ZINC003861213	0.227	ZINC028957444	0.242
ZINC100051405	0.216	ZINC003917708	0.236
ZINC003795098	0.213	ZINC003780800	0.233
ZINC040165222	0.212	ZINC003977803	0.233
ZINC004098512	0.211	ZINC004245665	0.233
ZINC003978654	0.211	ZINC150338767	0.233
ZINC005599377	0.210	ZINC261494649	0.231
ZINC003918137	0.209	ZINC004099104	0.230
ZINC004099035	0.208	ZINC004099008	0.228
ZINC095862725	0.207	ZINC008219992	0.227
ZINC100051402	0.205	ZINC256445970	0.225
ZINC100051398	0.205	ZINC034114798	0.225
ZINC018279854	0.202	ZINC003938704	0.223
ZINC011616152	0.200	ZINC256445978	0.223
ZINC100054977	0.200	ZINC100054168	0.222
ZINC004095762	0.198	ZINC095618881	0.221
ZINC033903112	0.194	ZINC150338908	0.220
ZINC034114798	0.193	ZINC410428646	0.219
ZINC256310200	0.192	ZINC003831231	0.219
ZINC053255716	0.191	ZINC040165255	0.218
ZINC040165217	0.191	ZINC095618817	0.218
ZINC261494611	0.191	ZINC060183860	0.217
ZINC001078624	0.190	ZINC031544842	0.216
ZINC036401969	0.190	ZINC028232755	0.215
ZINC004096846	0.189	ZINC008214470	0.215
ZINC013831818	0.188	ZINC095862725	0.214
ZINC100054975	0.187	ZINC003938684	0.213
ZINC003869855	0.187	ZINC096006020	0.213
ZINC261494644	0.186	ZINC410428645	0.212
ZINC100054746	0.184	ZINC015263894	0.212
ZINC003812887	0.184	ZINC013515301	0.212
ZINC040165202	0.183	ZINC169369935	0.211

ZINC031415836	0.183	ZINC095618643	0.210
ZINC261494634	0.183	ZINC261494651	0.210
ZINC150374458	0.183	ZINC095617635	0.209
ZINC256315095	0.183	ZINC004099035	0.209
ZINC003869685	0.183	ZINC003918087	0.208
ZINC102136963	0.183	ZINC095618644	0.208
ZINC102136971	0.183	ZINC256445975	0.207
ZINC013515302	0.183	ZINC003920266	0.207
ZINC031425361	0.183	ZINC094303245	0.207
ZINC085552699	0.182	ZINC100014909	0.206
ZINC261494599	0.182	ZINC043207238	0.206
ZINC261494594	0.182	ZINC150374458	0.205
ZINC021981188	0.182	ZINC100053657	0.205
ZINC040165247	0.182	ZINC095618877	0.205
ZINC005733652	0.182	ZINC100054212	0.204
ZINC100051400	0.182	ZINC095618866	0.204
ZINC004096817	0.181	ZINC001490477	0.204
ZINC100054183	0.180	ZINC040165217	0.204
ZINC040165218	0.180	ZINC150338755	0.202
ZINC077293633	0.180	ZINC100054194	0.202
ZINC011616153	0.180	ZINC000897256	0.201
ZINC100051411	0.180	ZINC095618878	0.201
ZINC100053131	0.180	ZINC043100709	0.201
ZINC002018621	0.179	ZINC003918428	0.200
ZINC003983907	0.179	ZINC256445982	0.200
ZINC013546270	0.179	ZINC003964325	0.200
ZINC003861133	0.179	ZINC100054214	0.200
ZINC095618628	0.179	ZINC261494576	0.200
ZINC100030679	0.179	ZINC261494563	0.198
ZINC003834173	0.179	ZINC085536956	0.198
ZINC031415834	0.179	ZINC049918330	0.198
ZINC100051419	0.178	ZINC095618641	0.198
ZINC012496345	0.178	ZINC003830994	0.197
ZINC008551674	0.178	ZINC003871889	0.197
ZINC031425077	0.177	ZINC003916214	0.197
ZINC000039906	0.177	ZINC000538658	0.196
ZINC100054096	0.177	ZINC018516586	0.196
ZINC239165117	0.176	ZINC052509366	0.196
ZINC077291461	0.176	ZINC095618642	0.196
ZINC003956771	0.176	ZINC000537805	0.196
ZINC261494610	0.176	ZINC003978083	0.195
ZINC004245625	0.175	ZINC148723177	0.195
ZINC095618750	0.175	ZINC029571072	0.195
ZINC012493465	0.175	ZINC028232746	0.195

ZINC077291459	0.175	ZINC085432544	0.194
ZINC150374443	0.175	ZINC077300530	0.194
ZINC239165118	0.175	ZINC072206342	0.194
ZINC003977803	0.175	ZINC077292462	0.194
ZINC261494667	0.174	ZINC004824235	0.194
ZINC005159763	0.174	ZINC003918453	0.194
ZINC002539702	0.174	ZINC014768469	0.194
ZINC256315101	0.174	ZINC150338912	0.193
ZINC022060265	0.173	ZINC000020252	0.193
ZINC000895390	0.173	ZINC003938686	0.193
ZINC012494987	0.173	ZINC000057319	0.192
ZINC012496781	0.173	ZINC256630457	0.192
ZINC028232755	0.173	ZINC100054205	0.191
ZINC003871576	0.173	ZINC077293633	0.191
ZINC095618921	0.173	ZINC100054198	0.190

The top 100 hits via each method are merged together and listed here.

Table S3. Selected drugs from the 2D similarity search using CVT-10216 as a reference compound.

ZINC ID	MACCS keys Similarity	RDkit Similarity	ECFP Similarity	FCFP Similarity
ZINC011616759	0.742	0.331	0.301	0.466
ZINC003812923	0.742	0.327	0.331	0.479
ZINC095616937	0.697	0.399	0.331	0.465
ZINC003831490	0.690	0.307	0.274	0.387
ZINC095618786	0.667	0.371	0.285	0.350
ZINC049933061	0.663	0.415	0.301	0.327
ZINC238730526	0.662	0.305	0.081	0.063
ZINC238730525	0.662	0.305	0.081	0.063
ZINC238730524	0.662	0.305	0.081	0.063
ZINC238730527	0.662	0.305	0.081	0.063
ZINC000538163	0.652	0.299	0.258	0.388
ZINC095618878	0.650	0.369	0.256	0.315
ZINC095618879	0.650	0.369	0.256	0.315
ZINC003813061	0.649	0.332	0.237	0.317
ZINC000597588	0.646	0.394	0.185	0.261
ZINC001842686	0.646	0.394	0.185	0.261
ZINC005140767	0.646	0.379	0.205	0.282
ZINC000538538	0.644	0.288	0.232	0.262
ZINC004617749	0.643	0.285	0.269	0.360
ZINC000896717	0.639	0.368	0.290	0.357
ZINC021298097	0.636	0.323	0.252	0.349
ZINC000057255	0.635	0.318	0.224	0.264
ZINC000020253	0.635	0.318	0.224	0.264

ZINC000002094	0.632	0.297	0.180	0.258
ZINC150347080	0.627	0.382	0.229	0.308
ZINC000049142	0.625	0.188	0.197	0.259
ZINC034049000	0.625	0.344	0.226	0.252
ZINC001538857	0.624	0.367	0.238	0.336
ZINC005140766	0.622	0.368	0.164	0.200
ZINC000002097	0.622	0.243	0.187	0.248
ZINC013233295	0.613	0.269	0.189	0.258
ZINC021981364	0.613	0.352	0.192	0.200
ZINC003812930	0.613	0.339	0.207	0.304
ZINC002040778	0.608	0.169	0.195	0.286
ZINC030691736	0.608	0.340	0.268	0.277
ZINC051133897	0.608	0.376	0.189	0.298
ZINC095892888	0.607	0.374	0.161	0.242
ZINC001536931	0.600	0.266	0.204	0.269
ZINC001482077	0.600	0.337	0.256	0.233
ZINC002019996	0.600	0.266	0.204	0.269
ZINC005423072	0.600	0.396	0.162	0.215
ZINC034104937	0.596	0.360	0.181	0.259
ZINC001481831	0.595	0.359	0.200	0.261
ZINC022054044	0.595	0.380	0.167	0.288
ZINC034303655	0.595	0.289	0.237	0.237
ZINC000596731	0.595	0.294	0.341	0.373
ZINC169289767	0.595	0.341	0.186	0.258
ZINC000001718	0.594	0.256	0.131	0.160
ZINC090693835	0.593	0.377	0.171	0.230
ZINC100051344	0.593	0.377	0.171	0.230
ZINC100051339	0.593	0.377	0.171	0.230
ZINC253476804	0.593	0.377	0.171	0.230
ZINC253476806	0.593	0.377	0.171	0.230
ZINC253476805	0.593	0.377	0.171	0.230
ZINC100006429	0.593	0.382	0.158	0.242
ZINC000035804	0.592	0.309	0.210	0.261
ZINC000002092	0.590	0.262	0.213	0.276
ZINC003806104	0.588	0.263	0.171	0.211
ZINC031707000	0.586	0.308	0.216	0.164
ZINC001535101	0.585	0.351	0.191	0.215
ZINC012503102	0.585	0.365	0.158	0.222
ZINC013129998	0.585	0.374	0.164	0.268
ZINC003833880	0.585	0.351	0.191	0.215
ZINC000537805	0.581	0.298	0.224	0.232
ZINC000056721	0.581	0.265	0.191	0.292
ZINC052509366	0.580	0.400	0.285	0.391
ZINC003955219	0.578	0.312	0.212	0.220

ZINC000896755	0.575	0.332	0.206	0.244
ZINC000601254	0.575	0.332	0.206	0.244
ZINC000897229	0.575	0.317	0.133	0.160
ZINC000897232	0.575	0.317	0.133	0.160
ZINC100022637	0.575	0.364	0.273	0.304
ZINC013761818	0.571	0.320	0.265	0.295
ZINC001842744	0.571	0.327	0.184	0.268
ZINC001842746	0.571	0.327	0.184	0.268
ZINC000607726	0.571	0.327	0.184	0.268
ZINC001530694	0.571	0.262	0.196	0.276
ZINC001842745	0.571	0.327	0.184	0.268
ZINC100054527	0.570	0.312	0.205	0.229
ZINC100054519	0.570	0.312	0.205	0.229
ZINC095551509	0.568	0.382	0.146	0.152
ZINC003809192	0.568	0.293	0.224	0.232
ZINC022047714	0.568	0.270	0.207	0.261
ZINC006091878	0.567	0.215	0.176	0.176
ZINC100015491	0.565	0.393	0.156	0.238
ZINC000897222	0.564	0.338	0.194	0.248
ZINC000601301	0.564	0.338	0.194	0.248
ZINC000000305	0.564	0.298	0.203	0.262
ZINC001846476	0.564	0.298	0.203	0.262
ZINC001530621	0.564	0.334	0.109	0.153
ZINC095619120	0.563	0.318	0.205	0.229
ZINC095619124	0.563	0.318	0.205	0.229
ZINC885764928	0.562	0.375	0.190	0.232
ZINC203757351	0.562	0.375	0.190	0.232
ZINC001690324	0.561	0.279	0.250	0.316
ZINC100016058	0.558	0.381	0.265	0.313
ZINC049089868	0.558	0.377	0.156	0.219
ZINC001542594	0.558	0.350	0.168	0.243
ZINC001846165	0.558	0.350	0.168	0.243
ZINC005783849	0.557	0.261	0.139	0.191
ZINC004098610	0.463	0.588	0.372	0.416
ZINC000895390	0.356	0.574	0.336	0.459
ZINC003872928	0.377	0.532	0.286	0.457
ZINC000001342	0.446	0.520	0.328	0.459
ZINC005733652	0.475	0.505	0.222	0.305
ZINC003872582	0.450	0.490	0.312	0.426
ZINC003977803	0.493	0.490	0.172	0.263
ZINC004098512	0.493	0.490	0.172	0.263
ZINC003871576	0.400	0.486	0.242	0.284
ZINC018185774	0.419	0.485	0.208	0.258
ZINC034114798	0.453	0.477	0.199	0.341

ZINC095862725	0.451	0.472	0.156	0.222
ZINC085552699	0.451	0.472	0.156	0.222
ZINC003869685	0.419	0.471	0.205	0.244
ZINC000608382	0.443	0.466	0.229	0.331
ZINC072206342	0.485	0.465	0.160	0.235
ZINC004096846	0.485	0.465	0.160	0.235
ZINC095537874	0.415	0.459	0.146	0.241
ZINC001530604	0.431	0.450	0.288	0.353
ZINC001530605	0.431	0.450	0.288	0.353
ZINC001530788	0.431	0.449	0.253	0.333
ZINC000056654	0.369	0.434	0.159	0.248
ZINC015919406	0.494	0.434	0.354	0.432
ZINC003919581	0.527	0.433	0.247	0.340
ZINC000000928	0.458	0.432	0.205	0.342
ZINC005159763	0.468	0.431	0.280	0.356
ZINC008214470	0.408	0.428	0.138	0.182
ZINC085432549	0.421	0.427	0.140	0.165
ZINC094303244	0.411	0.427	0.136	0.174
ZINC094303245	0.411	0.427	0.136	0.174
ZINC085432544	0.415	0.427	0.137	0.167
ZINC085555528	0.415	0.427	0.137	0.167
ZINC085537078	0.406	0.427	0.137	0.168
ZINC085536958	0.411	0.427	0.150	0.171
ZINC087496429	0.411	0.427	0.150	0.171
ZINC085536956	0.411	0.427	0.150	0.171
ZINC000057674	0.322	0.427	0.233	0.375
ZINC004099104	0.437	0.424	0.177	0.256
ZINC001542146	0.494	0.424	0.318	0.411
ZINC197178353	0.299	0.424	0.095	0.079
ZINC169746603	0.299	0.424	0.095	0.079
ZINC169987861	0.299	0.424	0.095	0.079
ZINC261494566	0.363	0.423	0.109	0.090
ZINC261494620	0.382	0.422	0.106	0.088
ZINC195835734	0.310	0.421	0.095	0.095
ZINC150338708	0.412	0.421	0.104	0.167
ZINC011616153	0.519	0.421	0.191	0.277
ZINC011616152	0.519	0.421	0.191	0.277
ZINC011616154	0.519	0.421	0.191	0.277
ZINC014879999	0.519	0.421	0.191	0.277
ZINC003831231	0.519	0.421	0.191	0.277
ZINC334138264	0.369	0.420	0.112	0.089
ZINC169621215	0.369	0.420	0.112	0.089
ZINC253848650	0.369	0.420	0.112	0.089
ZINC001612996	0.452	0.418	0.168	0.234

ZINC030731084	0.436	0.417	0.170	0.243
ZINC003875273	0.363	0.417	0.128	0.176
ZINC203686879	0.407	0.417	0.193	0.293
ZINC022058728	0.446	0.417	0.183	0.258
ZINC299818016	0.433	0.415	0.109	0.086
ZINC100378061	0.390	0.414	0.165	0.247
ZINC095618775	0.400	0.413	0.079	0.123
ZINC169621209	0.379	0.413	0.131	0.193
ZINC169621210	0.379	0.413	0.131	0.193
ZINC685933138	0.443	0.412	0.100	0.118
ZINC169621228	0.443	0.412	0.100	0.118
ZINC261527202	0.443	0.412	0.100	0.118
ZINC003826253	0.396	0.411	0.123	0.208
ZINC004212626	0.393	0.411	0.142	0.128
ZINC003985346	0.376	0.410	0.115	0.208
ZINC001550477	0.551	0.410	0.359	0.387
ZINC169621200	0.458	0.410	0.133	0.188
ZINC253975480	0.458	0.410	0.133	0.188
ZINC334138310	0.458	0.410	0.133	0.188
ZINC052955754	0.392	0.410	0.213	0.207
ZINC053683151	0.379	0.409	0.152	0.140
ZINC169621223	0.429	0.409	0.105	0.125
ZINC685933137	0.429	0.409	0.105	0.125
ZINC253947047	0.429	0.409	0.105	0.125
ZINC002015981	0.396	0.409	0.126	0.214
ZINC936069425	0.448	0.409	0.197	0.294
ZINC000538273	0.416	0.409	0.130	0.231
ZINC000537891	0.416	0.409	0.130	0.231
ZINC019701769	0.372	0.409	0.133	0.133
ZINC004099013	0.440	0.408	0.168	0.243
ZINC085537142	0.469	0.408	0.140	0.135
ZINC001611274	0.433	0.407	0.162	0.234
ZINC003607120	0.391	0.407	0.128	0.228
ZINC038197764	0.391	0.407	0.128	0.228
ZINC095626782	0.405	0.407	0.155	0.155
ZINC100051419	0.460	0.407	0.250	0.345
ZINC030691556	0.448	0.407	0.102	0.155
ZINC030691561	0.448	0.407	0.102	0.155
ZINC030691552	0.448	0.407	0.102	0.155
ZINC030691548	0.448	0.407	0.102	0.155
ZINC100051411	0.467	0.407	0.250	0.368
ZINC100051415	0.467	0.407	0.250	0.368
ZINC014880004	0.357	0.407	0.153	0.140
ZINC095564694	0.386	0.407	0.061	0.097

ZINC067664978	0.450	0.353	0.326	0.408
ZINC003782818	0.469	0.362	0.322	0.432
ZINC000598390	0.476	0.370	0.317	0.425
ZINC053084692	0.316	0.218	0.303	0.336
ZINC040899447	0.521	0.315	0.302	0.388
ZINC000538275	0.398	0.374	0.297	0.371
ZINC084668739	0.494	0.359	0.294	0.353
ZINC026005230	0.486	0.300	0.294	0.347
ZINC000007455	0.537	0.231	0.292	0.281
ZINC068202099	0.393	0.349	0.291	0.394
ZINC000608204	0.391	0.285	0.290	0.413
ZINC014210642	0.465	0.385	0.289	0.363
ZINC070466416	0.449	0.306	0.286	0.435
ZINC100014909	0.365	0.351	0.286	0.278
ZINC011679756	0.456	0.371	0.285	0.370
ZINC113149554	0.425	0.298	0.284	0.397
ZINC000001748	0.288	0.181	0.280	0.330
ZINC100051402	0.468	0.403	0.280	0.356
ZINC000579472	0.417	0.289	0.280	0.356
ZINC100051400	0.468	0.403	0.280	0.356
ZINC095618887	0.457	0.255	0.278	0.343
ZINC005761797	0.519	0.303	0.277	0.372
ZINC026007998	0.449	0.306	0.276	0.350
ZINC001585847	0.296	0.245	0.275	0.304
ZINC095618865	0.440	0.256	0.274	0.338
ZINC000000865	0.508	0.280	0.274	0.393
ZINC003612929	0.271	0.288	0.272	0.395
ZINC038726563	0.325	0.245	0.271	0.299
ZINC013761819	0.545	0.343	0.271	0.341
ZINC095618749	0.524	0.395	0.270	0.348
ZINC095618748	0.524	0.395	0.270	0.348
ZINC001544683	0.410	0.331	0.270	0.350
ZINC012503187	0.317	0.370	0.267	0.369
ZINC044699444	0.301	0.225	0.267	0.315
ZINC001895505	0.365	0.349	0.266	0.318
ZINC002020110	0.478	0.136	0.265	0.233
ZINC001530886	0.293	0.357	0.265	0.349
ZINC022060265	0.557	0.401	0.265	0.315
ZINC095618793	0.389	0.404	0.265	0.364
ZINC003932191	0.389	0.404	0.265	0.364
ZINC095618792	0.389	0.404	0.265	0.364
ZINC059719687	0.389	0.404	0.265	0.364
ZINC095617636	0.494	0.401	0.264	0.341
ZINC095617635	0.494	0.401	0.264	0.341

ZINC001493878	0.427	0.298	0.264	0.348
ZINC013531944	0.446	0.305	0.262	0.252
ZINC012341529	0.446	0.305	0.262	0.252
ZINC003873921	0.508	0.208	0.261	0.338
ZINC022056306	0.295	0.226	0.261	0.308
ZINC003817152	0.469	0.309	0.260	0.324
ZINC000006694	0.535	0.288	0.260	0.384
ZINC003818726	0.543	0.237	0.260	0.336
ZINC100051398	0.468	0.406	0.260	0.345
ZINC100051405	0.468	0.406	0.260	0.345
ZINC100015190	0.543	0.395	0.260	0.341
ZINC100015192	0.543	0.395	0.260	0.341
ZINC021981222	0.287	0.317	0.259	0.327
ZINC005888085	0.548	0.364	0.259	0.306
ZINC000902197	0.296	0.228	0.257	0.304
ZINC095618646	0.386	0.298	0.256	0.322
ZINC095618645	0.386	0.298	0.256	0.322
ZINC019632618	0.292	0.318	0.256	0.322
ZINC000001003	0.468	0.302	0.256	0.389
ZINC003831317	0.403	0.265	0.256	0.296
ZINC000119632	0.329	0.291	0.256	0.317
ZINC022060259	0.557	0.394	0.256	0.305
ZINC095618750	0.549	0.396	0.256	0.315
ZINC014952767	0.311	0.345	0.255	0.319
ZINC001565262	0.435	0.380	0.254	0.350
ZINC001481815	0.405	0.326	0.254	0.341
ZINC000601275	0.451	0.339	0.254	0.309
ZINC003830321	0.371	0.206	0.250	0.393
ZINC003975663	0.463	0.347	0.252	0.390
ZINC014210876	0.410	0.371	0.222	0.384
ZINC064033452	0.407	0.389	0.250	0.381
ZINC000057522	0.400	0.241	0.248	0.381
ZINC000125047	0.274	0.293	0.213	0.370
ZINC003920719	0.356	0.399	0.246	0.368
ZINC011615928	0.356	0.399	0.246	0.368
ZINC011615927	0.356	0.399	0.246	0.368
ZINC011615926	0.356	0.399	0.246	0.368
ZINC002017901	0.485	0.345	0.241	0.364
ZINC003812958	0.157	0.204	0.204	0.364
ZINC003871723	0.157	0.204	0.204	0.364
ZINC003916214	0.398	0.366	0.218	0.362
ZINC003816292	0.443	0.341	0.204	0.362
ZINC000006990	0.256	0.305	0.223	0.360
ZINC000021067	0.256	0.305	0.223	0.360

ZINC000002042	0.443	0.350	0.250	0.360
ZINC030691754	0.373	0.319	0.247	0.358
ZINC000000797	0.439	0.287	0.185	0.356
ZINC030691797	0.314	0.328	0.211	0.354
ZINC049643479	0.292	0.283	0.198	0.353
ZINC084589076	0.452	0.376	0.244	0.350
ZINC100006264	0.452	0.376	0.244	0.350
ZINC000001427	0.238	0.201	0.235	0.349
ZINC011616882	0.342	0.290	0.224	0.346
ZINC003932194	0.380	0.403	0.243	0.346
ZINC003816287	0.342	0.290	0.224	0.346
ZINC000001995	0.329	0.326	0.206	0.345
ZINC100030679	0.426	0.374	0.224	0.342
ZINC100030674	0.426	0.374	0.224	0.342

The top 100 hits via each method are merged together and listed here.

Table S4. Selected drugs from the 3D similarity search using CVT-10216 as a reference compound.

E3FP Similarity		USRCAT Score	
ZINC ID	Similarity	ZINC ID	Score
ZINC030691736	0.179	ZINC011616152	0.190
ZINC000001342	0.176	ZINC150601177	0.188
ZINC001530604	0.175	ZINC000968278	0.172
ZINC015919406	0.174	ZINC150588351	0.172
ZINC095618750	0.173	ZINC003872806	0.170
ZINC004098610	0.171	ZINC014879999	0.168
ZINC095618593	0.171	ZINC100090021	0.167
ZINC000895390	0.170	ZINC003872807	0.165
ZINC000003877	0.170	ZINC068204830	0.162
ZINC000621853	0.170	ZINC169289386	0.161
ZINC000000928	0.167	ZINC195761836	0.156
ZINC003812923	0.165	ZINC261106254	0.152
ZINC001658782	0.165	ZINC022058728	0.150
ZINC014969354	0.162	ZINC003872805	0.148
ZINC003919581	0.162	ZINC031544846	0.147
ZINC000105216	0.161	ZINC011616153	0.147
ZINC001550477	0.160	ZINC011616154	0.144
ZINC005421253	0.160	ZINC031544850	0.144
ZINC000403533	0.157	ZINC003872566	0.142
ZINC001844627	0.156	ZINC008214644	0.142
ZINC100054219	0.155	ZINC001530604	0.141
ZINC095618660	0.154	ZINC085552699	0.140
ZINC000000063	0.154	ZINC003831231	0.137
ZINC001530605	0.154	ZINC000538275	0.137

ZINC001542146	0.153	ZINC003824921	0.137
ZINC100054252	0.153	ZINC085536932	0.136
ZINC000056654	0.153	ZINC150339055	0.135
ZINC000005608	0.153	ZINC095618645	0.135
ZINC002572820	0.152	ZINC068206930	0.135
ZINC033994719	0.152	ZINC095618662	0.134
ZINC002382375	0.152	ZINC000538658	0.133
ZINC100015190	0.152	ZINC085537068	0.133
ZINC000607790	0.152	ZINC100022637	0.133
ZINC030691556	0.152	ZINC011615927	0.132
ZINC030691548	0.152	ZINC261494650	0.132
ZINC001530788	0.151	ZINC085537142	0.132
ZINC095617669	0.151	ZINC004099009	0.132
ZINC004215736	0.151	ZINC068202099	0.131
ZINC000057731	0.151	ZINC003775140	0.131
ZINC022060265	0.150	ZINC256630463	0.131
ZINC003918453	0.150	ZINC085537053	0.131
ZINC100054849	0.150	ZINC014210876	0.131
ZINC005699865	0.150	ZINC685933138	0.130
ZINC005159763	0.150	ZINC169621228	0.130
ZINC100015192	0.149	ZINC261527202	0.130
ZINC002021931	0.149	ZINC004099104	0.130
ZINC100051339	0.149	ZINC095617639	0.130
ZINC253476805	0.149	ZINC261494658	0.129
ZINC000001300	0.149	ZINC084758235	0.129
ZINC095616600	0.148	ZINC004074875	0.129
ZINC000000973	0.147	ZINC026824305	0.128
ZINC000002094	0.147	ZINC004393164	0.127
ZINC000005823	0.147	ZINC085555528	0.127
ZINC261494575	0.146	ZINC261494651	0.127
ZINC000601275	0.146	ZINC004098512	0.127
ZINC100053678	0.146	ZINC085536956	0.127
ZINC000968328	0.145	ZINC011677894	0.125
ZINC253476804	0.145	ZINC095618744	0.125
ZINC261494596	0.145	ZINC004824235	0.125
ZINC037868226	0.145	ZINC113149554	0.125
ZINC011616852	0.145	ZINC028639340	0.125
ZINC000057734	0.144	ZINC077313075	0.124
ZINC037475297	0.144	ZINC169621209	0.124
ZINC022047714	0.144	ZINC096006020	0.124
ZINC005957009	0.144	ZINC003938746	0.124
ZINC000089688	0.144	ZINC053683148	0.124
ZINC005733652	0.144	ZINC004215257	0.124
ZINC100051400	0.144	ZINC003991624	0.124

ZINC003875872	0.144	ZINC261139420	0.124
ZINC002019996	0.144	ZINC150338771	0.123
ZINC095618661	0.144	ZINC150347080	0.123
ZINC008214658	0.144	ZINC003830432	0.123
ZINC005179119	0.143	ZINC004474682	0.123
ZINC095618598	0.143	ZINC257362202	0.123
ZINC261494678	0.143	ZINC094566092	0.123
ZINC000002097	0.143	ZINC261494652	0.123
ZINC001565262	0.143	ZINC094303245	0.122
ZINC000598852	0.143	ZINC261106252	0.122
ZINC003975663	0.143	ZINC150338767	0.122
ZINC003813061	0.143	ZINC001612996	0.121
ZINC003869855	0.143	ZINC242548690	0.121
ZINC001656906	0.143	ZINC003934128	0.121
ZINC003871891	0.143	ZINC085432549	0.121
ZINC100053131	0.143	ZINC169369935	0.121
ZINC000608085	0.143	ZINC169621210	0.121
ZINC049933061	0.142	ZINC169368439	0.121
ZINC014879992	0.142	ZINC026985532	0.121
ZINC261494565	0.142	ZINC006716957	0.121
ZINC261494597	0.142	ZINC001895505	0.120
ZINC005166438	0.142	ZINC002528509	0.120
ZINC100015470	0.142	ZINC003799072	0.119
ZINC000621893	0.142	ZINC095617651	0.119
ZINC004216730	0.142	ZINC001530605	0.119
ZINC100051398	0.142	ZINC085537014	0.119
ZINC261494568	0.142	ZINC008234405	0.118
ZINC100054189	0.141	ZINC003995616	0.118
ZINC253476806	0.140	ZINC261494656	0.118
ZINC118912516	0.140	ZINC256015222	0.118
ZINC038929224	0.140	ZINC085537078	0.118
ZINC100051405	0.140	ZINC261494659	0.118

The top 100 hits via each method are merged together and listed here.

Table S5. Selected drugs from the 2D similarity search using CHEMBL114083 as a reference compound.

ZINC ID	MACCS keys Similarity	RDkit Similarity	ECFP Similarity	FCFP Similarity
ZINC095862725	0.809	0.441	0.243	0.347
ZINC085552699	0.809	0.441	0.243	0.347
ZINC013831819	0.762	0.165	0.163	0.180
ZINC003977786	0.756	0.275	0.177	0.267
ZINC003977787	0.756	0.275	0.177	0.267
ZINC004098610	0.739	0.684	0.500	0.650

ZINC003860441	0.717	0.392	0.176	0.349
ZINC036520252	0.706	0.288	0.199	0.320
ZINC072206342	0.702	0.439	0.172	0.347
ZINC004096846	0.702	0.439	0.172	0.347
ZINC001698306	0.690	0.153	0.194	0.286
ZINC000039092	0.682	0.261	0.193	0.291
ZINC000058117	0.674	0.258	0.188	0.275
ZINC003977803	0.673	0.502	0.185	0.363
ZINC004098512	0.673	0.502	0.185	0.363
ZINC095604030	0.673	0.273	0.122	0.149
ZINC003978654	0.667	0.323	0.144	0.233
ZINC001530604	0.667	0.460	0.239	0.354
ZINC001530788	0.667	0.468	0.205	0.333
ZINC001530605	0.667	0.460	0.239	0.354
ZINC013831818	0.659	0.156	0.174	0.200
ZINC000608382	0.655	0.415	0.213	0.364
ZINC000001342	0.652	0.664	0.304	0.515
ZINC100053131	0.651	0.280	0.268	0.340
ZINC100054755	0.647	0.307	0.113	0.231
ZINC100054746	0.647	0.307	0.113	0.231
ZINC100054749	0.647	0.307	0.113	0.231
ZINC095618817	0.647	0.307	0.113	0.231
ZINC100051419	0.644	0.376	0.233	0.383
ZINC003800475	0.640	0.249	0.135	0.176
ZINC005513434	0.640	0.249	0.135	0.176
ZINC003819138	0.640	0.273	0.233	0.314
ZINC013449462	0.636	0.222	0.178	0.252
ZINC000119983	0.636	0.261	0.168	0.275
ZINC013449465	0.636	0.222	0.178	0.252
ZINC013449409	0.636	0.223	0.178	0.252
ZINC013449412	0.636	0.223	0.178	0.252
ZINC005733652	0.636	0.569	0.235	0.406
ZINC095618751	0.636	0.348	0.233	0.349
ZINC031544837	0.635	0.189	0.232	0.269
ZINC040165255	0.635	0.277	0.110	0.194
ZINC003799072	0.635	0.186	0.235	0.273
ZINC040165217	0.635	0.277	0.110	0.194
ZINC031544850	0.635	0.189	0.232	0.269
ZINC040165218	0.635	0.277	0.110	0.194
ZINC003785268	0.635	0.186	0.235	0.273
ZINC031544846	0.635	0.189	0.232	0.269
ZINC031544842	0.635	0.189	0.232	0.269
ZINC003938684	0.635	0.307	0.117	0.227
ZINC000538275	0.633	0.333	0.314	0.406

ZINC064622163	0.630	0.173	0.062	0.118
ZINC004098633	0.630	0.259	0.223	0.336
ZINC003869685	0.628	0.511	0.226	0.343
ZINC018185774	0.628	0.557	0.230	0.363
ZINC005834731	0.625	0.234	0.250	0.336
ZINC003799319	0.625	0.229	0.228	0.300
ZINC005834799	0.625	0.234	0.250	0.336
ZINC102136963	0.623	0.297	0.103	0.186
ZINC008234405	0.623	0.297	0.103	0.186
ZINC102136971	0.623	0.297	0.103	0.186
ZINC040165221	0.623	0.297	0.103	0.186
ZINC004096817	0.623	0.298	0.104	0.189
ZINC013515302	0.623	0.298	0.104	0.189
ZINC256310200	0.623	0.298	0.104	0.189
ZINC008551674	0.623	0.298	0.104	0.189
ZINC040165220	0.623	0.297	0.103	0.186
ZINC004099009	0.623	0.310	0.133	0.268
ZINC077291459	0.623	0.288	0.126	0.211
ZINC012496781	0.623	0.298	0.104	0.189
ZINC004099008	0.623	0.310	0.133	0.268
ZINC040165219	0.623	0.297	0.103	0.186
ZINC150374443	0.623	0.298	0.104	0.189
ZINC013515304	0.623	0.298	0.104	0.189
ZINC077291461	0.623	0.288	0.126	0.211
ZINC013515303	0.623	0.298	0.104	0.189
ZINC000518554	0.622	0.242	0.209	0.317
ZINC100051402	0.622	0.375	0.321	0.423
ZINC100051398	0.622	0.377	0.276	0.396
ZINC005159763	0.622	0.406	0.298	0.410
ZINC100051405	0.622	0.377	0.276	0.396
ZINC100051400	0.622	0.375	0.321	0.423
ZINC008143614	0.621	0.244	0.060	0.097
ZINC002509755	0.621	0.255	0.248	0.350
ZINC002528486	0.621	0.255	0.248	0.350
ZINC033903112	0.620	0.274	0.135	0.243
ZINC118915219	0.618	0.223	0.051	0.095
ZINC261494618	0.618	0.223	0.051	0.095
ZINC118915221	0.618	0.223	0.051	0.095
ZINC261494617	0.618	0.223	0.051	0.095
ZINC261494616	0.618	0.223	0.051	0.095
ZINC118915220	0.618	0.223	0.051	0.095
ZINC118915218	0.618	0.223	0.051	0.095
ZINC261494619	0.618	0.223	0.051	0.095
ZINC002528510	0.617	0.263	0.276	0.368

ZINC002526388	0.617	0.263	0.276	0.368
ZINC002526389	0.617	0.263	0.276	0.368
ZINC095618646	0.617	0.322	0.286	0.380
ZINC002528509	0.617	0.263	0.276	0.368
ZINC095618645	0.617	0.322	0.286	0.380
ZINC004099035	0.615	0.276	0.111	0.197
ZINC000895390	0.512	0.735	0.337	0.472
ZINC003872928	0.500	0.654	0.270	0.454
ZINC003871576	0.571	0.572	0.317	0.412
ZINC000057674	0.463	0.504	0.224	0.365
ZINC034114798	0.596	0.467	0.182	0.420
ZINC003872582	0.568	0.442	0.277	0.382
ZINC000056654	0.479	0.433	0.165	0.276
ZINC000057731	0.468	0.414	0.233	0.296
ZINC015919406	0.449	0.408	0.255	0.317
ZINC001542146	0.449	0.388	0.238	0.308
ZINC095537874	0.510	0.386	0.120	0.226
ZINC100051411	0.591	0.377	0.233	0.383
ZINC100051415	0.591	0.377	0.233	0.383
ZINC003872446	0.578	0.374	0.137	0.237
ZINC002548959	0.489	0.369	0.187	0.283
ZINC000000928	0.359	0.364	0.165	0.306
ZINC011616153	0.394	0.363	0.170	0.307
ZINC011616152	0.394	0.363	0.170	0.307
ZINC011616154	0.394	0.363	0.170	0.307
ZINC014879999	0.394	0.363	0.170	0.307
ZINC003831231	0.394	0.363	0.170	0.307
ZINC000057734	0.500	0.358	0.165	0.277
ZINC033986664	0.614	0.355	0.288	0.375
ZINC022060265	0.444	0.354	0.270	0.384
ZINC033986666	0.614	0.350	0.277	0.362
ZINC003869855	0.578	0.349	0.226	0.310
ZINC100015190	0.429	0.348	0.234	0.354
ZINC100015192	0.429	0.348	0.234	0.354
ZINC005599377	0.614	0.348	0.312	0.388
ZINC100030679	0.568	0.347	0.226	0.343
ZINC100030674	0.568	0.347	0.226	0.343
ZINC022060259	0.444	0.347	0.260	0.372
ZINC095618750	0.460	0.346	0.220	0.360
ZINC002007481	0.492	0.345	0.149	0.299
ZINC000000063	0.492	0.345	0.149	0.299
ZINC001565262	0.511	0.342	0.207	0.352
ZINC084589076	0.600	0.341	0.217	0.352
ZINC100006264	0.600	0.341	0.217	0.352

ZINC003782807	0.475	0.338	0.132	0.252
ZINC000538285	0.525	0.337	0.169	0.288
ZINC197178353	0.188	0.337	0.095	0.089
ZINC169746603	0.188	0.337	0.095	0.089
ZINC169987861	0.188	0.337	0.095	0.089
ZINC003919581	0.247	0.337	0.164	0.259
ZINC334138264	0.430	0.336	0.093	0.113
ZINC169621215	0.430	0.336	0.093	0.113
ZINC253848650	0.430	0.336	0.093	0.113
ZINC195835734	0.200	0.335	0.095	0.113
ZINC203686879	0.410	0.335	0.137	0.215
ZINC150338708	0.451	0.335	0.094	0.202
ZINC004099104	0.507	0.334	0.148	0.285
ZINC685933138	0.452	0.334	0.086	0.145
ZINC169621228	0.452	0.334	0.086	0.145
ZINC261527202	0.452	0.334	0.086	0.145
ZINC261494620	0.447	0.334	0.102	0.117
ZINC261494566	0.440	0.334	0.090	0.115
ZINC085432549	0.500	0.334	0.122	0.186
ZINC085537078	0.481	0.333	0.114	0.184
ZINC003875273	0.472	0.333	0.107	0.211
ZINC008214470	0.481	0.333	0.124	0.193
ZINC085536958	0.487	0.332	0.121	0.187
ZINC087496429	0.487	0.332	0.121	0.187
ZINC085536956	0.487	0.332	0.121	0.187
ZINC169621223	0.435	0.332	0.091	0.154
ZINC685933137	0.435	0.332	0.091	0.154
ZINC253947047	0.435	0.332	0.091	0.154
ZINC094303244	0.506	0.332	0.128	0.196
ZINC094303245	0.506	0.332	0.128	0.196
ZINC100378061	0.476	0.332	0.143	0.290
ZINC085432544	0.513	0.332	0.123	0.188
ZINC085555528	0.513	0.332	0.123	0.188
ZINC004245665	0.554	0.331	0.153	0.270
ZINC001612996	0.539	0.331	0.133	0.252
ZINC022058728	0.513	0.330	0.146	0.279
ZINC030731084	0.519	0.330	0.156	0.246
ZINC003830212	0.516	0.328	0.209	0.292
ZINC043194409	0.481	0.225	0.389	0.414
ZINC043207237	0.448	0.215	0.342	0.377
ZINC145808877	0.448	0.215	0.342	0.377
ZINC002570857	0.359	0.225	0.314	0.290
ZINC006030312	0.375	0.244	0.304	0.343
ZINC006037085	0.375	0.244	0.304	0.343

ZINC001895505	0.556	0.323	0.298	0.356
ZINC002034516	0.556	0.211	0.294	0.363
ZINC015263894	0.521	0.276	0.293	0.327
ZINC001544683	0.597	0.313	0.293	0.392
ZINC000001748	0.415	0.127	0.289	0.330
ZINC095618715	0.471	0.233	0.286	0.366
ZINC095618714	0.471	0.233	0.286	0.366
ZINC001571009	0.400	0.227	0.281	0.312
ZINC021981363	0.413	0.248	0.281	0.384
ZINC021981361	0.413	0.248	0.281	0.384
ZINC003991624	0.607	0.218	0.274	0.303
ZINC044699444	0.453	0.219	0.272	0.347
ZINC003831317	0.500	0.208	0.271	0.293
ZINC001537194	0.608	0.154	0.270	0.292
ZINC022056306	0.431	0.221	0.266	0.339
ZINC000057278	0.491	0.127	0.265	0.288
ZINC000003911	0.491	0.127	0.265	0.288
ZINC003860453	0.511	0.248	0.264	0.308
ZINC084758479	0.478	0.289	0.263	0.384
ZINC000902197	0.403	0.224	0.262	0.333
ZINC022056370	0.435	0.224	0.258	0.325
ZINC022056375	0.435	0.224	0.258	0.325
ZINC026005230	0.260	0.252	0.258	0.336
ZINC095617672	0.438	0.308	0.258	0.372
ZINC100054019	0.333	0.249	0.257	0.303
ZINC001530775	0.434	0.170	0.256	0.288
ZINC000968326	0.400	0.244	0.254	0.319
ZINC000968327	0.400	0.244	0.254	0.319
ZINC095618710	0.478	0.237	0.254	0.366
ZINC002017901	0.481	0.274	0.254	0.308
ZINC095618711	0.478	0.237	0.254	0.366
ZINC003869608	0.465	0.167	0.252	0.288
ZINC002041017	0.324	0.226	0.252	0.284
ZINC001606505	0.324	0.226	0.252	0.284
ZINC000537874	0.476	0.202	0.252	0.294
ZINC095618858	0.427	0.252	0.250	0.384
ZINC095618857	0.427	0.252	0.250	0.384
ZINC001550766	0.522	0.234	0.250	0.322
ZINC095617649	0.600	0.268	0.248	0.357
ZINC095617650	0.600	0.268	0.248	0.357
ZINC001540640	0.600	0.270	0.248	0.357
ZINC006019305	0.600	0.270	0.248	0.357
ZINC038726563	0.460	0.236	0.248	0.317
ZINC095617674	0.444	0.314	0.248	0.361

ZINC019632628	0.449	0.258	0.248	0.309
ZINC011677901	0.525	0.240	0.247	0.293
ZINC011677894	0.525	0.240	0.247	0.293
ZINC011677890	0.525	0.240	0.247	0.293
ZINC019364224	0.435	0.170	0.246	0.287
ZINC019364222	0.435	0.170	0.246	0.287
ZINC008214629	0.609	0.212	0.246	0.261
ZINC000001290	0.333	0.152	0.245	0.305
ZINC000056544	0.333	0.152	0.245	0.305
ZINC000056549	0.356	0.151	0.245	0.305
ZINC000056546	0.356	0.151	0.245	0.305
ZINC000056547	0.356	0.151	0.245	0.305
ZINC003873160	0.299	0.271	0.244	0.282
ZINC095618866	0.524	0.280	0.244	0.329
ZINC095618867	0.524	0.280	0.244	0.329
ZINC001319967	0.370	0.204	0.243	0.388
ZINC003201958	0.370	0.204	0.243	0.388
ZINC000000407	0.370	0.204	0.243	0.388
ZINC011616526	0.370	0.204	0.243	0.388
ZINC009302317	0.370	0.204	0.243	0.388
ZINC003830970	0.370	0.204	0.243	0.388
ZINC043207566	0.500	0.279	0.128	0.452
ZINC001546066	0.492	0.269	0.231	0.410
ZINC000001320	0.476	0.277	0.218	0.373
ZINC003872687	0.508	0.237	0.230	0.372
ZINC000607910	0.508	0.237	0.230	0.372
ZINC002019929	0.456	0.267	0.208	0.360
ZINC005760515	0.464	0.221	0.191	0.356
ZINC005760453	0.464	0.221	0.191	0.356
ZINC068202099	0.356	0.318	0.209	0.356
ZINC000608359	0.520	0.255	0.206	0.355
ZINC001534965	0.500	0.298	0.217	0.355
ZINC000968328	0.373	0.247	0.234	0.354
ZINC000968330	0.373	0.247	0.234	0.354
ZINC013682481	0.412	0.307	0.179	0.352
ZINC003975663	0.522	0.297	0.172	0.351
ZINC000607872	0.500	0.256	0.200	0.350
ZINC002014976	0.500	0.256	0.200	0.350
ZINC000002042	0.407	0.301	0.233	0.349
ZINC000608172	0.373	0.207	0.226	0.345

The top 100 hits via each method are merged together and listed here.

Table S6. Selected drugs from the 3D similarity search using CHEMBL114083 as a reference compound.

E3FP Similarity		USRCAT Score	
ZINC ID	Similarity	ZINC ID	Score
ZINC000895390	0.215	ZINC011616152	0.170
ZINC004098610	0.215	ZINC014879999	0.145
ZINC034636383	0.185	ZINC150588351	0.140
ZINC095618750	0.184	ZINC261106254	0.133
ZINC003869685	0.180	ZINC150601177	0.128
ZINC005733652	0.167	ZINC000968278	0.126
ZINC000896463	0.165	ZINC100090021	0.120
ZINC000057731	0.163	ZINC256015224	0.116
ZINC001530788	0.161	ZINC022058728	0.115
ZINC022060265	0.160	ZINC169289386	0.114
ZINC033986664	0.158	ZINC150338703	0.114
ZINC095618751	0.158	ZINC261494658	0.113
ZINC095862725	0.157	ZINC238850855	0.113
ZINC001530604	0.155	ZINC004074875	0.110
ZINC004098512	0.154	ZINC003872806	0.110
ZINC049783754	0.154	ZINC068204830	0.110
ZINC003812923	0.154	ZINC256015225	0.109
ZINC013831818	0.154	ZINC004393164	0.109
ZINC000001684	0.153	ZINC011615927	0.109
ZINC000056654	0.152	ZINC011616153	0.108
ZINC100051402	0.152	ZINC003872807	0.108
ZINC030691548	0.151	ZINC085552699	0.107
ZINC002548959	0.149	ZINC000538275	0.107
ZINC003978654	0.147	ZINC261494581	0.106
ZINC253613241	0.146	ZINC410428644	0.106
ZINC000003877	0.146	ZINC261494650	0.106
ZINC033986666	0.146	ZINC257362202	0.105
ZINC261494677	0.146	ZINC261494659	0.105
ZINC003780898	0.146	ZINC003872566	0.105
ZINC095617651	0.145	ZINC011616154	0.104
ZINC003871576	0.144	ZINC085555528	0.104
ZINC015263894	0.144	ZINC261494626	0.104
ZINC001542146	0.144	ZINC169368439	0.104
ZINC000001342	0.143	ZINC261494652	0.103
ZINC003782818	0.143	ZINC003824921	0.103
ZINC001530605	0.143	ZINC150338819	0.103
ZINC256445970	0.143	ZINC261494647	0.102
ZINC018185774	0.143	ZINC049637509	0.102
ZINC060183860	0.142	ZINC685933138	0.101
ZINC030691556	0.142	ZINC169621228	0.101
ZINC014969354	0.142	ZINC261527202	0.101
ZINC095618715	0.142	ZINC095618795	0.101

ZINC261494678	0.141	ZINC003872805	0.101
ZINC000039092	0.141	ZINC169369935	0.101
ZINC100051415	0.141	ZINC085537078	0.101
ZINC003869855	0.141	ZINC008234405	0.101
ZINC261494565	0.141	ZINC256015222	0.100
ZINC002572820	0.140	ZINC255977093	0.100
ZINC100051398	0.140	ZINC238730530	0.100
ZINC003875483	0.140	ZINC261106252	0.100
ZINC100051419	0.140	ZINC008220175	0.100
ZINC005599377	0.140	ZINC169362009	0.100
ZINC085589133	0.139	ZINC204073689	0.100
ZINC015919406	0.138	ZINC095618645	0.100
ZINC005760515	0.138	ZINC095862733	0.100
ZINC001895505	0.138	ZINC001612996	0.100
ZINC000037293	0.138	ZINC003831231	0.099
ZINC000621853	0.138	ZINC195761836	0.099
ZINC100015192	0.138	ZINC003830432	0.099
ZINC100054849	0.137	ZINC014210876	0.099
ZINC005699865	0.137	ZINC096006012	0.099
ZINC058581064	0.137	ZINC261139420	0.099
ZINC072206342	0.137	ZINC004099104	0.099
ZINC085552699	0.137	ZINC004098512	0.099
ZINC002018621	0.136	ZINC261494651	0.099
ZINC095618850	0.136	ZINC000538658	0.098
ZINC100054856	0.136	ZINC028639340	0.098
ZINC003860441	0.136	ZINC068206930	0.098
ZINC000001098	0.136	ZINC100053654	0.098
ZINC001698306	0.136	ZINC150338771	0.098
ZINC003871832	0.136	ZINC085536956	0.098
ZINC023358248	0.135	ZINC001530604	0.098
ZINC039472045	0.135	ZINC002528509	0.097
ZINC095618623	0.135	ZINC085432549	0.097
ZINC022056375	0.135	ZINC068202099	0.097
ZINC000403533	0.135	ZINC004474682	0.097
ZINC003872446	0.134	ZINC256015223	0.097
ZINC100017736	0.134	ZINC150338767	0.097
ZINC002522694	0.134	ZINC095618880	0.097
ZINC002509755	0.134	ZINC003978083	0.097
ZINC003137600	0.133	ZINC245190611	0.097
ZINC004096846	0.133	ZINC252286876	0.097
ZINC031425076	0.133	ZINC040165220	0.097
ZINC000085742	0.133	ZINC238850853	0.097
ZINC085537026	0.133	ZINC011677894	0.096
ZINC003831172	0.133	ZINC072190231	0.096

ZINC100006264	0.133	ZINC008214644	0.096
ZINC003872928	0.133	ZINC085537068	0.096
ZINC005442135	0.132	ZINC256640979	0.096
ZINC031495147	0.132	ZINC169621209	0.096
ZINC095617649	0.132	ZINC150338755	0.096
ZINC100051400	0.131	ZINC026824305	0.096
ZINC000389747	0.131	ZINC094566092	0.096
ZINC100051411	0.130	ZINC003831120	0.096
ZINC000000063	0.130	ZINC230122970	0.095
ZINC038929225	0.130	ZINC004099008	0.095
ZINC100053131	0.130	ZINC085537142	0.095
ZINC100015190	0.130	ZINC009212428	0.095
ZINC004245625	0.130	ZINC094303245	0.095
ZINC000607790	0.130	ZINC113149554	0.095

The top 100 hits via each method are merged together and listed here.

Table S7. Hit details using different method/reference combinations for the compounds with $\Delta E_{dock} \leq$ -10 kcal/mol.

ZINC ID	Name	*q*	2D				3D	
			MACCS Keys	RDKit	ECFP4	FCFP4	E3FP	USRCAT
ZINC011679756	Eltrombopag	-3	N/N/N	N/N/N	N/Y/N	N/Y/N	N/N/N	N/N/N
ZINC049783754	Indacaterol-8-O-Glucuronide	0	N/N/N	Y/N/N	Y/N/N	Y/N/N	N/N/Y	N/N/N
ZINC011679756	Eltrombopag	-2	N/N/N	N/N/N	N/Y/N	N/Y/N	N/N/N	N/N/N
ZINC001542146	Pranlukast-IA	-1	N/N/N	N/Y/Y	N/Y/N	N/Y/N	N/Y/Y	N/N/N
ZINC001542146	Pranlukast-IB	-1	N/N/N	N/Y/Y	N/Y/N	N/Y/N	N/Y/Y	N/N/N
ZINC095618662	ZINC095618662	1	N/N/N	N/N/N	N/N/N	N/N/N	N/N/N	N/Y/N
ZINC019632618	Imatinib-I	1	N/N/N	N/N/N	N/Y/N	N/N/N	N/N/N	N/N/N
ZINC019632618	Imatinib-II	1	N/N/N	N/N/N	N/Y/N	N/N/N	N/N/N	N/N/N
ZINC003824921	Fexofenadine-I	0	N/N/N	N/N/N	N/N/N	N/N/N	N/N/N	N/Y/Y
ZINC021981222	N-Desmethyl Imatinib	1	N/N/N	N/N/N	N/Y/N	N/N/N	N/N/N	N/N/N
ZINC150339055	ZINC150339055	1	N/N/N	N/N/N	N/N/N	N/N/N	N/N/N	N/Y/N
ZINC008220175	Zeaxanthin	0	N/N/N	N/N/N	N/N/N	N/N/N	N/N/N	N/N/Y
ZINC077313075	Sorafenib Beta-D-Glucuronide	-1	N/N/N	N/N/N	N/N/N	N/N/N	N/N/N	N/Y/N
ZINC113149554	Netarsudil	0	N/N/N	N/N/N	N/Y/N	N/Y/N	N/N/N	N/Y/Y
ZINC001493878	Sorafenib	0	N/N/N	N/N/N	N/Y/N	N/Y/N	N/N/N	N/N/N
ZINC000968278	Troglitazone	0	N/N/N	N/N/N	N/N/N	N/N/N	N/N/N	N/Y/Y
ZINC000968278	Troglitazone	-1	N/N/N	N/N/N	N/N/N	N/N/N	N/N/N	N/Y/Y
ZINC013449462	5-O-Desmethyldonepezil-I	0	N/N/Y	N/N/N	N/N/N	N/N/N	N/N/N	N/N/N
ZINC003872566	Fexofenadine-II	0	N/N/N	N/N/N	N/N/N	N/N/N	N/N/N	N/Y/Y
ZINC000057674	Flavone	0	N/N/N	N/Y/Y	N/N/N	Y/Y/Y	N/N/N	N/N/N
ZINC000021067	R Sarizotan	1	N/N/N	N/N/N	N/N/N	N/Y/N	N/N/N	N/N/N
ZINC006037085	(R)-4'-Hydroxyflurbipron	-1	N/N/N	N/N/N	N/N/Y	N/N/Y	N/N/N	N/N/N

ZINC068202099	Erismodegib	0	N/N/N	N/N/N	N/Y/N	N/Y/Y	N/N/N	N/Y/Y
ZINC003817152	Sorafenib N-Oxide	0	N/N/N	N/N/N	N/Y/N	N/N/N	N/N/N	N/N/N
ZINC000896717	Accolate	-1	N/Y/N	N/N/N	N/Y/N	N/Y/N	N/N/N	N/N/N
ZINC001550477	Lapatinib	1	N/N/N	N/Y/N	N/Y/N	N/Y/N	N/Y/N	N/N/N
ZINC013515303	17-Alpha-Estradiol-3-Glucuronide	-1	N/N/Y	Y/N/N	N/N/N	N/N/N	N/N/N	N/N/N
ZINC015919406	Pranlukast-IIA	-1	N/N/N	N/Y/Y	N/Y/Y	N/Y/N	N/Y/Y	N/N/N
ZINC013449412	6-O-Desmethyldonepeil	0	N/N/Y	N/N/N	N/N/N	N/N/N	N/N/N	N/N/N
ZINC013449412	6-O-Desmethyldonepeil	1	N/N/Y	N/N/N	N/N/N	N/N/N	N/N/N	N/N/N
ZINC006030312	(S)-4'-Hydroxyflurbipron	-1	N/N/N	N/N/N	N/N/Y	N/N/Y	N/N/N	N/N/N
ZINC113149554	Netarsudil	1	N/N/N	N/N/N	N/Y/N	N/Y/N	N/N/N	N/Y/Y
ZINC015919406	Pranlukast-IIB	-1	N/N/N	N/Y/Y	N/Y/Y	N/Y/N	N/Y/Y	N/N/N
ZINC013449462	5-O-Desmethyldonepezil-I	1	N/N/Y	N/N/N	N/N/N	N/N/N	N/N/N	N/N/N
ZINC013449465	5-O-Desmethyldonepezil-II	0	N/N/Y	N/N/N	N/N/N	N/N/N	N/N/N	N/N/N
ZINC000006990	S Sarizotan	1	N/N/N	N/N/N	N/N/N	N/Y/N	N/N/N	N/N/N
ZINC000105216	Naproxen	-1	N/N/N	N/N/N	N/N/N	N/N/N	N/Y/N	N/N/N
ZINC256630457	ZINC256630457	1	N/N/N	N/N/N	N/N/N	N/N/N	N/N/N	Y/N/N
ZINC256630463	ZINC256630463	1	N/N/N	N/N/N	N/N/N	N/N/N	N/N/N	N/Y/N
ZINC028639340	Posaconazole	0	N/N/N	N/N/N	N/N/N	N/N/N	N/N/N	N/Y/Y
ZINC026985532	Sequinavir	0	N/N/N	N/N/N	N/N/N	N/N/N	N/N/N	N/Y/N
ZINC026985532	Sequinavir	1	N/N/N	N/N/N	N/N/N	N/N/N	N/N/N	N/Y/N

"Y" and “N” indicate whether the compounds can be hit using difference 2D/3D methods with three reference molecules of daidzin/CVT-10216/CHEMBL114083 or not.

Table S8. Root-mean-square deviations (RMSDs) of non-hydrogen atoms of ligands from the initial configurations (i.e. the docking poses).

Ligand	*q*	ChainA	ChainB	ChainC	ChainD
Sequinavir	1	0.16 ± 0.01	0.20 ± 0.01	0.38 ± 0.02	0.35 ± 0.02
R Sarizotan	1	0.16 ± 0.01	0.20 ± 0.03	0.22 ± 0.02	0.21 ± 0.01
S Sarizotan	1	0.17 ± 0.02	0.15 ± 0.02	0.20 ± 0.02	0.16 ± 0.04
Netarsudil	1	0.17 ± 0.02	0.18 ± 0.01	0.22 ± 0.02	0.22 ± 0.02
Zeaxanthin	0	0.32 ± 0.01	0.33 ± 0.04	0.30 ± 0.02	0.37 ± 0.01
Troglitazone	0	0.14 ± 0.01	0.13 ± 0.02	0.14 ± 0.05	0.20 ± 0.01
Sequinavir	0	0.32 ± 0.02	0.24 ± 0.03	0.24 ± 0.02	0.24 ± 0.02
Netarsudil	0	0.16 ± 0.04	0.22 ± 0.04	0.16 ± 0.02	0.16 ± 0.01
Fexofenadine-II	0	0.13 ± 0.01	0.20 ± 0.02	0.20 ± 0.02	0.13 ± 0.02
Troglitazone	-1	0.21 ± 0.01	0.13 ± 0.02	0.11 ± 0.02	0.16 ± 0.01
Pranlukast-IA	-1	0.28 ± 0.03	0.08 ± 0.01	0.29 ± 0.02	0.30 ± 0.02
Pranlukast-IIB	-1	0.20 ± 0.03	0.18 ± 0.02	0.23 ± 0.02	0.16 ± 0.03
Pranlukast-IIA	-1	0.19 ± 0.01	0.23 ± 0.02	0.19 ± 0.02	0.22 ± 0.01
Pranlukast-IB	-1	0.27 ± 0.04	0.29 ± 0.04	0.33 ± 0.02	0.29 ± 0.02
Naproxen	-1	0.14 ± 0.01	0.14 ± 0.00	0.09 ± 0.01	0.14 ± 0.01
Daidzin	0	0.13 ± 0.01	0.12 ± 0.01	0.13 ± 0.01	0.13 ± 0.01
CVT-10216	0	0.20 ± 0.01	0.09 ± 0.02	0.09 ± 0.01	0.14 ± 0.02
CHEMBL114083	0	0.22 ± 0.03	0.20 ± 0.04	0.20 ± 0.03	0.17 ± 0.02

Table S9. Energy decomposition (kcal/mol) of identified key residues for binding with neutral inhibitors of Netarsudil, Troglitazone, and Zeaxanthin.

Residue	Netarsudil (q = 0)				Troglitazone (q = 0)				Zeaxanthin (q = 0)			
	ΔE_{MM}	ΔG_{polar}	$\Delta G_{nonpolar}$	ΔE_{bind}	ΔE_{MM}	ΔG_{polar}	$\Delta G_{nonpolar}$	ΔE_{bind}	ΔE_{MM}	ΔG_{polar}	$\Delta G_{nonpolar}$	ΔE_{bind}
Lys112	0.61 ± 0.02	0.12 ± 0.03	0.00 ± 0.00	0.73 ± 0.03	-0.16 ± 0.02	0.83 ± 0.05	0.00 ± 0.00	0.68 ± 0.05	0.01 ± 0.01	1.18 ± 0.03	0.00 ± 0.00	1.19 ± 0.03
Val115	-0.53 ± 0.02	0.32 ± 0.03	-0.06 ± 0.00	-0.27 ± 0.02	-0.01 ± 0.00	-0.03 ± 0.00	0.00 ± 0.00	-0.03 ± 0.00	-1.59 ± 0.03	0.33 ± 0.02	-0.19 ± 0.00	-1.44 ± 0.03
Ile116	-1.33 ± 0.03	0.21 ± 0.01	-0.11 ± 0.00	-1.23 ± 0.03	-0.08 ± 0.00	-0.04 ± 0.00	0.00 ± 0.00	-0.12 ± 0.00	-1.54 ± 0.04	-0.01 ± 0.01	-0.13 ± 0.00	-1.67 ± 0.04
Val120	-4.77 ± 0.06	1.71 ± 0.03	-0.37 ± 0.00	-3.44 ± 0.06	-1.77 ± 0.04	0.76 ± 0.05	-0.21 ± 0.00	-1.21 ± 0.04	-1.76 ± 0.02	0.19 ± 0.01	-0.21 ± 0.00	-1.79 ± 0.02
Asp121	-3.67 ± 0.04	3.26 ± 0.09	-0.03 ± 0.00	-0.45 ± 0.10	-0.99 ± 0.03	0.07 ± 0.17	-0.03 ± 0.00	-0.94 ± 0.15	-0.17 ± 0.01	-1.42 ± 0.03	0.00 ± 0.00	-1.59 ± 0.04
Asp123	-3.02 ± 0.12	2.63 ± 0.15	-0.07 ± 0.00	-0.45 ± 0.07	-0.25 ± 0.02	-0.67 ± 0.06	-0.01 ± 0.00	-0.92 ± 0.05	-0.11 ± 0.01	-1.00 ± 0.02	0.00 ± 0.00	-1.11 ± 0.02
Met124	-2.06 ± 0.03	0.25 ± 0.01	-0.13 ± 0.00	-1.94 ± 0.03	-1.33 ± 0.03	0.31 ± 0.01	-0.13 ± 0.00	-1.15 ± 0.03	-0.98 ± 0.04	0.39 ± 0.01	-0.15 ± 0.01	-0.74 ± 0.04
Lys127	-1.44 ± 0.12	4.77 ± 0.23	-0.10 ± 0.00	3.22 ± 0.15	0.05 ± 0.03	1.41 ± 0.12	-0.02 ± 0.00	1.44 ± 0.11	0.02 ± 0.01	0.89 ± 0.03	-0.00 ± 0.00	0.91 ± 0.03
Phe170	-0.82 ± 0.03	0.05 ± 0.01	-0.07 ± 0.00	-0.83 ± 0.03	-2.36 ± 0.04	0.69 ± 0.02	-0.18 ± 0.01	-1.84 ± 0.04	-2.80 ± 0.03	0.85 ± 0.01	-0.22 ± 0.00	-2.17 ± 0.04
Leu173	-1.03 ± 0.03	0.09 ± 0.01	-0.10 ± 0.00	-1.04 ± 0.03	-1.16 ± 0.02	0.23 ± 0.01	-0.09 ± 0.00	-1.03 ± 0.02	-0.89 ± 0.03	0.36 ± 0.01	-0.08 ± 0.00	-0.61 ± 0.03
Met174	-0.23 ± 0.02	0.11 ± 0.01	-0.02 ± 0.00	-0.14 ± 0.01	-1.15 ± 0.05	0.49 ± 0.02	-0.10 ± 0.00	-0.76 ± 0.03	-1.67 ± 0.03	0.63 ± 0.01	-0.08 ± 0.00	-1.12 ± 0.03
Trp177	-0.78 ± 0.02	0.60 ± 0.02	-0.07 ± 0.00	-0.25 ± 0.02	-1.72 ± 0.04	0.92 ± 0.03	-0.09 ± 0.00	-0.89 ± 0.03	-2.08 ± 0.03	1.05 ± 0.02	-0.12 ± 0.00	-1.15 ± 0.02
Thr244	-0.04 ± 0.00	0.01 ± 0.00	0.00 ± 0.00	-0.03 ± 0.00	-1.00 ± 0.02	-0.00 ± 0.01	-0.03 ± 0.00	-1.03 ± 0.02	-0.45 ± 0.02	0.18 ± 0.01	-0.05 ± 0.00	-0.32 ± 0.02
Phe296	-0.84 ± 0.02	0.37 ± 0.03	-0.09 ± 0.00	-0.56 ± 0.02	-1.41 ± 0.04	0.41 ± 0.01	-0.12 ± 0.00	-1.12 ± 0.04	-1.56 ± 0.06	0.79 ± 0.03	-0.18 ± 0.01	-0.95 ± 0.04
Arg329	0.17 ± 0.02	1.41 ± 0.13	-0.01 ± 0.00	1.56 ± 0.13	0.17 ± 0.02	0.40 ± 0.02	0.00 ± 0.00	0.56 ± 0.02	0.00 ± 0.02	0.87 ± 0.03	0.00 ± 0.00	0.87 ± 0.03
Glu340	-0.67 ± 0.04	0.28 ± 0.09	-0.05 ± 0.00	-0.43 ± 0.06	-0.13 ± 0.01	-0.27 ± 0.02	0.00 ± 0.00	-0.40 ± 0.01	-1.05 ± 0.04	-0.32 ± 0.06	-0.13 ± 0.00	-1.50 ± 0.05
Asp457	0.96 ± 0.05	-2.37 ± 0.07	-0.03 ± 0.00	-1.45 ± 0.05	-2.29 ± 0.06	2.83 ± 0.15	-0.20 ± 0.00	0.35 ± 0.11	-1.45 ± 0.04	3.93 ± 0.17	-0.20 ± 0.00	2.28 ± 0.15
Phe459	-2.42 ± 0.06	1.46 ± 0.05	-0.24 ± 0.01	-1.20 ± 0.03	-3.48 ± 0.06	1.44 ± 0.03	-0.29 ± 0.01	-2.34 ± 0.06	-2.69 ± 0.03	1.32 ± 0.02	-0.22 ± 0.00	-1.59 ± 0.04
NAD^+	-0.00 ± 0.00	-0.08 ± 0.00	0.00 ± 0.00	-0.08 ± 0.00	-2.46 ± 0.05	1.01 ± 0.02	-0.03 ± 0.00	-1.48 ± 0.05	-0.13 ± 0.02	0.14 ± 0.06	-0.00 ± 0.00	0.01 ± 0.04

Table S10. Energy decomposition (kcal/mol) of identified key residues for binding with Netarsudil (q = +1) and Sequinavir (q = +1).

Residue	Netarsudil (q = 1)				Sequinavir (q = +1)			
	ΔE_{MM}	ΔG_{polar}	$\Delta G_{nonpolar}$	ΔE_{bind}	ΔE_{MM}	ΔG_{polar}	$\Delta G_{nonpolar}$	ΔE_{bind}
Ala7	4.76 ± 0.03	-0.04 ± 0.01	0.00 ± 0.00	4.72 ± 0.03	5.01 ± 0.03	0.00 ± 0.01	0.00 ± 0.00	5.01 ± 0.03
Glu96	-4.33 ± 0.02	0.03 ± 0.01	0.00 ± 0.00	-4.30 ± 0.02	-4.27 ± 0.02	0.01 ± 0.01	0.00 ± 0.00	-4.26 ± 0.01
Arg99	5.49 ± 0.02	-0.08 ± 0.01	0.00 ± 0.00	5.41 ± 0.02	5.52 ± 0.02	-0.11 ± 0.02	0.00 ± 0.00	5.41 ± 0.01
Glu106	-4.38 ± 0.01	0.21 ± 0.02	0.00 ± 0.00	-4.17 ± 0.02	-4.45 ± 0.01	0.46 ± 0.02	0.00 ± 0.00	-3.99 ± 0.02
Lys112	5.22 ± 0.02	-0.21 ± 0.03	0.00 ± 0.00	5.01 ± 0.03	5.42 ± 0.02	-0.98 ± 0.04	0.00 ± 0.00	4.44 ± 0.04
Asp121	-6.16 ± 0.03	0.38 ± 0.04	0.00 ± 0.00	-5.78 ± 0.03	-5.79 ± 0.03	0.84 ± 0.07	-0.00 ± 0.00	-4.95 ± 0.07
Asp123	-8.14 ± 0.06	0.18 ± 0.04	0.00 ± 0.00	-7.95 ± 0.05	-8.28 ± 0.05	0.84 ± 0.08	-0.01 ± 0.00	-7.44 ± 0.06
Lys127	8.74 ± 0.09	-0.18 ± 0.04	-0.00 ± 0.00	8.56 ± 0.06	8.60 ± 0.06	0.61 ± 0.15	-0.08 ± 0.01	9.12 ± 0.15
Arg130	4.58 ± 0.02	-0.07 ± 0.01	0.00 ± 0.00	4.51 ± 0.02	4.50 ± 0.01	-0.01 ± 0.01	0.00 ± 0.00	4.49 ± 0.01
Lys178	4.03 ± 0.02	-0.15 ± 0.02	0.00 ± 0.00	3.88 ± 0.01	4.43 ± 0.01	-0.85 ± 0.03	0.00 ± 0.00	3.58 ± 0.03
Glu268	-3.87 ± 0.02	0.11 ± 0.02	0.00 ± 0.00	-3.76 ± 0.01	-5.29 ± 0.02	1.99 ± 0.11	-0.01 ± 0.00	-3.30 ± 0.09
Asp282	-5.04 ± 0.02	0.41 ± 0.01	0.00 ± 0.00	-4.63 ± 0.02	-4.70 ± 0.02	0.38 ± 0.01	0.00 ± 0.00	-4.32 ± 0.01
Asp284	-4.63 ± 0.02	0.31 ± 0.01	0.00 ± 0.00	-4.31 ± 0.01	-4.68 ± 0.04	0.35 ± 0.01	0.00 ± 0.00	-4.33 ± 0.03
Glu288	-8.41 ± 0.05	1.19 ± 0.03	-0.00 ± 0.00	-7.22 ± 0.05	-7.31 ± 0.05	1.73 ± 0.09	-0.04 ± 0.00	-5.62 ± 0.06
Arg325	5.90 ± 0.03	-0.72 ± 0.02	0.00 ± 0.00	5.18 ± 0.02	5.83 ± 0.02	-0.78 ± 0.02	-0.00 ± 0.00	5.05 ± 0.02
Arg329	5.63 ± 0.03	2.91 ± 0.12	-0.03 ± 0.00	8.51 ± 0.11	6.26 ± 0.03	-1.29 ± 0.02	0.00 ± 0.00	4.97 ± 0.02
Glu340	-7.15 ± 0.04	2.77 ± 0.07	-0.10 ± 0.00	-4.48 ± 0.06	-6.03 ± 0.03	0.57 ± 0.01	0.00 ± 0.00	-5.46 ± 0.03
Glu476	-4.10 ± 0.02	0.27 ± 0.02	0.00 ± 0.00	-3.83 ± 0.01	-4.17 ± 0.01	0.55 ± 0.02	0.00 ± 0.00	-3.62 ± 0.02
NAD+	-2.37 ± 0.01	0.09 ± 0.01	0.00 ± 0.00	-2.28 ± 0.01	-2.71 ± 0.01	0.44 ± 0.02	0.00 ± 0.00	-2.27 ± 0.02

Table S11. Energy decomposition (kcal/mol) of identified key residues for binding with R Sarizotan (q = +1) and S Sarizotan (q = +1).

**Residue**	**R Sarizotan (q = +1)**				**S Sarizotan (q = +1)**			
	ΔE_{MM}	ΔG_{polar}	$\Delta G_{nonpolar}$	ΔE_{bind}	ΔE_{MM}	ΔG_{polar}	$\Delta G_{nonpolar}$	ΔE_{bind}
Ala7	4.49 ± 0.03	-0.15 ± 0.01	0.00 ± 0.00	4.34 ± 0.02	4.76 ± 0.04	-0.07 ± 0.01	0.00 ± 0.00	4.69 ± 0.03
Glu96	-4.57 ± 0.01	0.51 ± 0.01	0.00 ± 0.00	-4.06 ± 0.01	-3.94 ± 0.02	0.18 ± 0.01	0.00 ± 0.00	-3.76 ± 0.01
Arg99	6.27 ± 0.02	-1.54 ± 0.02	0.00 ± 0.00	4.74 ± 0.01	5.22 ± 0.02	-0.50 ± 0.02	0.00 ± 0.00	4.72 ± 0.01
Glu106	-6.66 ± 0.02	1.65 ± 0.03	0.00 ± 0.00	-5.01 ± 0.03	-5.33 ± 0.01	0.90 ± 0.02	0.00 ± 0.00	-4.43 ± 0.03
Lys112	8.02 ± 0.02	-2.34 ± 0.04	0.00 ± 0.00	5.68 ± 0.04	6.97 ± 0.02	-1.45 ± 0.04	0.00 ± 0.00	5.53 ± 0.05
Asp121	-12.17±0.06	5.86 ± 0.16	-0.01 ± 0.00	-6.32 ± 0.13	-7.75 ± 0.04	1.75 ± 0.06	-0.00 ± 0.00	-6.00 ± 0.06
Asp123	-9.40 ± 0.05	4.24 ± 0.10	-0.08 ± 0.00	-5.24 ± 0.07	-6.61 ± 0.05	0.55 ± 0.02	-0.00 ± 0.00	-6.07 ± 0.03
Lys127	8.33 ± 0.04	-1.45 ± 0.06	-0.02 ± 0.00	6.86 ± 0.08	6.68 ± 0.05	-0.40 ± 0.02	0.00 ± 0.00	6.28 ± 0.04
Arg130	5.04 ± 0.01	-0.73 ± 0.01	0.00 ± 0.00	4.30 ± 0.01	4.10 ± 0.01	-0.23 ± 0.01	0.00 ± 0.00	3.87 ± 0.01
Lys178	5.27 ± 0.02	-0.69 ± 0.04	0.00 ± 0.00	4.58 ± 0.04	4.14 ± 0.01	-0.39 ± 0.01	0.00 ± 0.00	3.75 ± 0.02
Glu268	-5.14 ± 0.02	0.53 ± 0.06	0.00 ± 0.00	-4.61 ± 0.06	-3.97 ± 0.01	0.38 ± 0.01	0.00 ± 0.00	-3.60 ± 0.01
Asp282	-3.97 ± 0.02	0.28 ± 0.01	0.00 ± 0.00	-3.69 ± 0.01	-5.09 ± 0.03	0.70 ± 0.02	0.00 ± 0.00	-4.39 ± 0.02
Asp284	-3.78 ± 0.02	0.23 ± 0.01	0.00 ± 0.00	-3.55 ± 0.02	-4.91 ± 0.03	0.62 ± 0.02	0.00 ± 0.00	-4.30 ± 0.02
Glu288	-5.50 ± 0.03	0.65 ± 0.01	0.00 ± 0.00	-4.85 ± 0.03	-8.70 ± 0.09	2.67 ± 0.07	-0.05 ± 0.00	-6.08 ± 0.05
Arg325	4.57 ± 0.04	-0.46 ± 0.01	0.00 ± 0.00	4.12 ± 0.03	6.45 ± 0.05	-1.38 ± 0.04	-0.00 ± 0.00	5.07 ± 0.02
Arg329	6.05 ± 0.03	-1.24 ± 0.02	0.00 ± 0.00	4.81 ± 0.02	7.88 ± 0.04	-2.02 ± 0.06	0.00 ± 0.00	5.86 ± 0.06
Glu340	-5.42 ± 0.03	0.67 ± 0.01	0.00 ± 0.00	-4.75 ± 0.03	-7.12 ± 0.08	1.32 ± 0.08	-0.01 ± 0.00	-5.79 ± 0.04
Glu476	-5.11 ± 0.01	0.87 ± 0.02	0.00 ± 0.00	-4.25 ± 0.02	-3.88 ± 0.01	0.38 ± 0.01	0.00 ± 0.00	-3.51 ± 0.01
NAD+	-3.14 ± 0.01	0.35 ± 0.01	0.00 ± 0.00	-2.79 ± 0.01	-2.57 ± 0.01	0.12 ± 0.01	0.00 ± 0.00	-2.45 ± 0.01
